# Microfluidic Nanosensor for Label-Free Multiplexed Detection of Breast Cancer Biomarkers via Surface-Enhanced Reflective FTIR Spectroscopy Using Thin Gold Films and Antibody-Oriented Gold Nanourchin: Feasibility Study

**DOI:** 10.3390/mi16111268

**Published:** 2025-11-11

**Authors:** Mohammad E. Khosroshahi, Gayathri Senthilchelvan, Victor Oyebolu

**Affiliations:** 1 Nanobiophotonics & Biomedical Research Laboratory, M.I.S. Electronics Inc., Richmond Hill, ON L4B 1B4, Canada; 2 Institute for Advanced Non-Destructive and Non-Invasive Diagnostic Technologies (IANDIT), University of Toronto, Toronto, ON M5S 3G8, Canada; 3 Department of Mechanical and Industrial Engineering, University of Toronto, Toronto, ON M5S 3G8, Canada; 4 Department of Biomedical Engineering, Toronto Metropolitan University, Toronto, ON M5B 2H3, Canada

**Keywords:** microfluidic sensor, surface-enhanced Raman scattering, breast cancer, biomarkers, Near FTIR, PCA statistical analysis

## Abstract

The simultaneous detection of multiple cancer biomarkers using microfluidic multiplexed immunosensors is gaining significant interest in the field of Point-of-Care diagnostics. This study highlights integrating surface-enhanced infrared Fourier transform (SE-FTIR) with a plasmonic-active nanostructure thin film (PANTF) on a printed circuit board (PCB), housed within a microfluidic device for rapid, non-destructive detection of breast cancer (BC). Detection uses monoclonal antibody (mAb)-functionalized gold nanourchins (GNUs) on dual sensing regions. A total of 12 serum samples (24 data points) were tested for HER-II and CA 15-3. The system demonstrated a SE-FTIR enhancement factor (EF) of ~0.18 × 10^5^ using Rhodamine 6G (R6G). Calibration with HER-II (1–100 ng/mL) and CA 15-3 (10–100 U/mL) showed linear responses (R^2^ = 0.8 and 0.76, respectively). Measurements of unknowns were performed at 1 µL/min over 68 min, with 43 min for biomarker interaction. SE-FTIR spectra were recorded at active zones and analyzed using SpectraView (SV), a custom Python 3.12-based tool. Data preprocessing included filtering (*SciPy’s filtfilt*) and baseline correction using the Improved Asymmetric Least Squares (*IASLS*) algorithm (*pybaselines.Whittaker*). Fourier cross-correlation (FCC) showed stronger signal consistency for HER-II. Partial Least Squares (PLS) regression, a dimensionality reduction technique, enabled clear discrimination between the samples and types, with classification accuracy reaching 1.0. Cancer staging based on these biomarkers yielded an overall accuracy of 0.54, indicating that classification regardless of biomarker type. Further studies involving larger and more diverse sample sets are critical before any definitive conclusions can be drawn.

## 1. Introduction

Breast cancer (BC) is the most common malignancy among women and the second leading cause of cancer-related death worldwide [1]. Particularly affecting developing countries [1,2], BC stands out due to its molecular heterogeneity, morphological and clinical characteristics, hence leading to complicated subtypes with distinct clinical outcomes and responses to treatment [3]. Based on the most recent global data from 2022, BC continues to be the most frequently diagnosed cancer worldwide. Data from the World Health Organization (WHO) and the World Cancer Research Fund International indicate that around 2.3 million women were diagnosed with BC globally in 2022, underscoring its status as the most prevalent cancer affecting women. That same year, an estimated 670,000 women died from BC, representing approximately 6.9% of all cancer-related deaths worldwide. Projections suggest that if current trends continue, by 2050, annual new cases of breast cancer will rise by 38% to reach 3.2 million, while the number of deaths is expected to increase by 68%, reaching an estimated 1.1 million annually.

The HER-II gene encodes the HER-II protein, a cell surface receptor expressed on breast epithelial cells that plays a critical role in regulating cell growth and proliferation. Overexpression of HER-II is one of the earliest molecular alterations observed in breast carcinogenesis and is regarded as a potential real-time indicator for both initial tumour detection and disease recurrence. Triple-negative breast cancer (TNBC) is defined by the lack of expression of human epidermal growth factor receptor 2 (HER-II), estrogen receptors (ER), and progesterone receptors (PR), which limits the effectiveness of receptor-targeted treatments and complicates both diagnosis and therapeutic intervention [3,4]. Cancer antigen 15-3 (CA 15-3) is a glycoprotein derived from the mucin (MUC) family—specifically, transmembrane proteins such as MUC1. Members of the MUC1 family, including CA 15-3, MCA, CA 549, and CA 27.29, are secreted by the luminal surface of glandular epithelial cells and have been investigated for their utility in early detection and prognostic assessment of BC in prediagnostic samples [5,6]. Malignant breast tumours exhibit increased expression of the MUC1 gene, which produces the CA 15-3 protein commonly used as a BC tumour marker [7]. The amount of CA 15-3 in the blood reflects the size of the tumour. Among breast cancer biomarkers, CA 15-3 is the most frequently utilized, often rising above 30 U/mL in patients with the disease [8]. Therefore, detecting CA 15-3 at very low levels could play a crucial role in the early diagnosis and effective monitoring of breast cancer.

Creating highly sensitive diagnostic methods for point-of-care (POC) use is crucial yet challenging, especially for early cancer detection. The demand for such tools is rising, driven by the goal to reduce unnecessary biopsies of benign tissues, which often cause physical pain, emotional distress, and higher medical expenses. The ideal diagnostic approach should be non-invasive, affordable, user-friendly, and able to provide fast and accurate results.

Several major drawbacks of current BC diagnostic techniques include their invasive nature, reliance on ionizing radiation and radioactive agents such as gadolinium, and incompatibility with patients who have pacemakers or breast implants. Moreover, these methods often demand costly, specialized facilities for handling radioisotopes, involve iodine-based contrast agents, and face limitations in sensitivity and spatial resolution. As a result, there is a risk of both false positive and false negative outcomes, which can significantly impact patients’ mental well-being, confidence, and morale.

Biosensors are characterized by their high sensitivity and selectivity, portability, compact design, minimal sample requirements, low signal-to-noise ratios, and capability for remote sensing applications [6]. Immunosensors, in particular, are distinguished by the specificity of antigen–antibody interactions, positioning them as promising candidates for the development of POC diagnostic platforms. These systems offer several advantages, including full automation, rapid response times, cost-effectiveness, high sensitivity, portability, and robust precision and accuracy [7,8]. Unlike conventional immunoassays, label-free (or direct) immunosensors can detect chemical or physical changes resulting directly from antigen–antibody interactions without the need for labelling agents. For effective early cancer detection, however, it remains critical to accurately identify and rapidly quantify cancer-specific biomarkers. Microfluidic technology plays a key role in this context by enabling precise control of fluid dynamics at the microscale within integrated, miniaturized POC devices. This facilitates rapid analyte mixing and reaction, thereby significantly enhancing the sensitivity and selectivity of biosensors [9,10,11]. Consequently, microfluidic chips have emerged as cost-effective alternatives to conventional diagnostic methods, offering advantages such as reduced sample volume, rapid analysis, seamless integration, portability, and suitability for on-site testing [12].

Blood-based biomarkers play a crucial role in non-invasive cancer screening and have the potential to significantly alleviate the overall economic burden on healthcare systems [13]. Early detection of these biomarkers, even at low concentrations, alongside detailed pathological characterization, can offer valuable insights into the initial stages of tumour development. This early identification is particularly beneficial for cancers such as BC, enabling diagnosis at a more treatable phase and thereby improving patient survival rates [14,15]. While biomarkers alone are not the definitive method for cancer diagnosis, they serve as important supplementary tools that enhance and support conventional diagnostic procedures.

When exposed to an external electric field, plasmonic nanoparticles (PNPs) exhibit a coherent collective oscillation of conduction electrons, resulting in pronounced optical absorption and scattering attributed to localized surface plasmon resonance (LSPR), which involves non-propagating surface plasmons [16]. Unlike LSPR, when the excitation frequency of incident electromagnetic radiation matches the resonance frequency of surface plasmons, strong coupling occurs at the metal–dielectric interface, enabling the excitation and propagation of collective electron oscillations along the interface. This phenomenon is termed propagating surface plasmon resonance or surface plasmon polaritons (SPPs). Among diverse PNPs, anisotropic three-dimensional nanocrystals such as GNU with branched and irregular surface morphologies exhibit distinctive optical responses compared to spherical gold nanoparticles of comparable core diameter. The complex geometry of GNUs, characterized by multiple sharp protrusions and high curvature regions, leads to a pronounced redshift in the LSPR peak due to altered electron oscillation dynamics. Each protruding spike functions as an individual plasmonic hotspot, supporting multiple LSPR modes that induce intense electromagnetic field confinement and enhancement within subwavelength volumes at the nanospike tips. This multi-modal plasmonic behaviour significantly amplifies near-field effects, enhancing sensitivity in applications such as surface-enhanced spectroscopies and nanoscale optical sensing [17].

The (TGF + GNU) system, i.e., plasmonic-active nanostructured thin film (PANTF) offers several advantages over individual NPs, including a stronger ability to generate enhanced electric fields and induce wavelength shifts [18,19,20]. This is largely due to the efficient coupling between SPs in the thin film and LSPR in the PNS, resulting in increased sensitivity. The interaction between LSPs and SPPs depends on the angle of incidence, primarily because SPP excitation requires wavevector (momentum) matching, which is angle-sensitive. The reproducibility of plasmonic hotspots formed between the thin film and PNS makes this configuration highly suitable for various plasmonic-based sensing applications, including propagating SP sensing, LSPR, SERS, and plasmon-induced organic reactions [17,21,22]. The PANTF system offers several key advantages for sensing applications. First, it is capable of simultaneously supporting both propagating SPPs and LSPR, enabling multimodal plasmonic interactions [23,24]. Second, the strong coupling between the SPPs of the thin film and the plasmonic modes of the nanostructures leads to significantly enhanced sensitivity. This synergistic effect amplifies the electromagnetic field intensity near the sensor surface, making the PANTF system an effective transducer for a wide range of plasmonic-based sensing modalities, including propagating SPP-based sensors, localized plasmonic resonance (PR) methods, SERS, and SE-FTIR-based techniques.

Immunosensors offer high sensitivity and specificity, enable detection at very low concentrations, and support multiplexed analysis in complex samples [25]. In label-free immunosensing, detection is achieved through the direct binding of target analytes to antibodies immobilized on the sensor surface. Among various analytical platforms, microfluidic systems as POC devices have emerged as powerful tools for biomarker detection and to precisely control fluids at the microscale due to their ability to handle small sample volumes, integrate multiple assay steps on a single chip, and provide rapid, high-throughput analysis [10]. Multiplexed immunosensors enable simultaneous detection of various biomarkers, not only increasing the information yield from a single patient sample but also enhancing diagnostic sensitivity and specificity by capturing the complex molecular signatures associated with different breast cancer subtypes. This approach enables rapid analysis, seamless integration, and portability, making it ideal for on-site testing. It improves clinical sensitivity and specificity, minimizes sample handling, preserves valuable specimens, and reduces both testing costs and turnaround time [11].

In this context, the PANTF-based immunosensor is particularly promising, as it combines the molecular specificity of immunoassays with the ultra-sensitivity of the platform. This integration is expected to provide a highly sensitive, selective, and rapid detection platform for various biomolecular targets, making it well-suited for advanced diagnostic applications, especially in cancer and infectious disease monitoring. The NIR region primarily features overtone and combination bands of fundamental vibrational modes, particularly those associated with functional groups in which a relatively heavy atom, such as carbon (C), nitrogen (N), oxygen (O), or sulfur (S), is bonded to a hydrogen atom [26]. NIR spectroscopy involves both electronic and vibrational transitions, with electronic transition bands typically appearing in the near-infrared region as generally weak spectral features.

Owing to its ability to generate distinct biochemical fingerprints, FT-NIR has become a reliable tool, particularly valuable for analyzing surface chemistries involved in the synthesis and fabrication of biosensors [27]. Conducting investigations at the nanoscale enhances the accuracy of NIR spectroscopy, as it involves small biomolecules that are well-suited for interaction with near-infrared light [28]. The NIR spectrum is commonly divided into three distinct regions. Region I (800–1200 nm or 12,500–8500 cm^−1^) encompass spectral bands associated with electronic transitions, first overtones, typically involving carbon-based bonds, and combination bands related to functional groups such as –CH, –OH, –NH, and –SH. Region II (1200–1800 nm or 8500–5500 cm^−1^) primarily includes the first overtones of stretching vibrations involving atoms like carbon, oxygen, and nitrogen, along with a range of combination modes arising from these vibrational transitions. This region is important for both quantitative and qualitative analysis and is especially useful in structural studies, including investigations of hydrogen bonding. Region III (1800–2500 nm or 5500–4000 cm^−1^) is dominated by combination bands arising from multiple vibrational modes. Overall, NIR spectroscopy encompasses both vibrational and electronic transitions, although the electronic bands in this region are typically weak.

Building on our recent studies involving the directional orientation of mAbs on thin gold films [29], colloidal GNU [30], and non-microfluidic PANTF with both single [31] and dual active detection areas [32] for biomarker detection, we propose to extend our research to (a) fabricate a dual PANTF-based microfluidic system to simultaneously detect BC biomarkers HER-II and CA15-3 binding to mAb in BCS using FT-NIR spectroscopy, (b) Build the calibration curves to quantify the biomarkers concentrations, and (c) To statistically analyze the data using PCA and Fourier cross-correlation (FCC).

## 2. Materials and Methods

### 2.1. Reagents

Citric acid–stabilized gold nanoparticles (GNU) with an average diameter of 90 nm were purchased from Cytodiagnostics (Burlington, ON, Canada). The supplied suspension contained 20 mL of nanoparticles at a concentration of 5.37 × 10^9^ particles/mL, with a mass concentration of 3.97 × 10^−2^ mg/mL and a molar concentration of 8.92 × 10^−12^ M, dispersed in 0.1 mM phosphate-buffered saline (PBS). Sodium phosphate (MilliporeSigma, Oakville, ON, Canada), pure ethanol reagent alcohol (MilliporeSigma, Oakville, ON, Canada), adipic acid dihydrazide (ADH) (MilliporeSigma, Oakville, ON, Canada), 1,6-hexanedithiol (HDT) (MilliporeSigma, Oakville, ON, Canada), EDC (MilliporeSigma, Oakville, ON, Canada), 2-(N-morpholino)ethanesulfonic acid hydrate (MES hydrate) (MilliporeSigma, Oakville, ON, Canada), sodium chloride (NaCl, MW = 58.44 g/mol) (MilliporeSigma, Oakville, ON, Canada), ethanolamine (NH_2_CH_2_CH_2_OH, 72068-100 mL, MW = 61.09 g/mol) (MilliporeSigma, Oakville, ON, Canada), 37% hydrochloric acid (HCl) (MilliporeSigma, Oakville, ON, Canada), sodium periodate (NaIO_4_) (MilliporeSigma, Oakville, ON, Canada), 30 kDA centrifugal filters (UFC503096, MilliporeSigma, Oakville, ON, Canada), microcentrifuge (OF-17710-11, Cole-Palmer, Mississauga, ON, Canada), micropipette tips (Cole-Palmer, Mississauga, ON, Canada), a combo pH meter (BLU2300E, Bluelab, Tauranga, New Zealand), and bovine serum albumin (BSA, A2153-10G, MilliporeSigma, Oakville, ON, Canada) were purchased. 1X PBS pH 7.4 (Fisher Scientific, Ottawa, ON, Canada), ultra-pure distilled water (Fisher Scientific, Ottawa, ON, Canada), and 70% ethanol in water (BP82031GAL, Fisher Scientific, Ottawa, ON, Canada), all analytical grade, were also purchased. 5k HS-PEG-COOH (PEG: polyethylene glycol) was purchased from (HE003019-5K, Biochempeg, Watertown, MA, USA). The microcentrifuge, vortex shaker (RK-04729-07), orbital shaker (RK-51700-13), and an analytical balance (Sartorius) were purchased from (Cole-Palmer, Mississauga, ON, Canada). In addition, scintillation vials (Grainger Inc., Caledon, ON, Canada), a fume hood (Mystaire Inc., Creedmoor, NC, USA), and ultrasonic bath (Elmasonic, Elma, Singen, Germany) were also used during the experiment. CA15-3 and HER-II standard solutions (i.e., pure) were purchased from (Lee Biosolutions, Maryland Heights, MO, USA) and (1129-ER-050, R&D Systems, Minneapolis, MN, USA), respectively. Serum samples from breast cancer (BCS) patients with known HER-II concentrations and CA15-3 status were purchased from both Precision for Medicine (Norton, MA, USA) and BioIVT (Hicksville, NY, USA). All chemicals and reagents were of analytical grade. HER-II and CA15-3 monoclonal antibodies (mAb) were purchased from SinoBiological (10004-MM03, HER-II/ErB_2_/CD340 Antibody, Mouse Mab, and 12123-MM05, MUC1/Mucin 1/CD227 Antibody, mouse Mab, Wayne, PA, USA), respectively. Table 1 summarizes the clinical specifications of the BCS samples, alongside their respective CA15-3 and HER-II statuses.

### 2.2. Cleaning of PCB

A 50 nm thin gold film (TGF) was coated on the PCB base by electroless deposition in a single-step bath immersion technique. The nickel component resists copper diffusion and protects against contamination. The final product is highly corrosion-resistant. Electroless deposition is the uniform coating of a metallic layer, such as gold, on a surface through chemical reduction in metal ions in an aqueous solution and the subsequent deposition of metal without using electricity. The bath produces a much tighter, lower porosity deposit. The substrates were placed within a 50 mL beaker containing 70% ethanol for cleaning before immobilization, functionalization and conjugation. The beaker was sealed and sonicated (Elma, Singen, Germany) for 30 min at 60 kHz and 60% power to completely clean the TGF. The PCB was rinsed with a fresh ethanol solution to remove any remaining debris and air-dried.

### 2.3. Preparation of 15 pM GNU

To prepare a 15 pM concentrated solution of 90 nm GNU, 8.92 pM of GNU was added to three different microcentrifuge tubes under a biosafety cabinet: 1.12 mL each to tubes I and II, and 1.13 mL to tube III. An additional tube containing 1.13 mL of water was prepared for balance. All tubes were centrifuged at 3200 rpm for 30 min. Following centrifugation, the supernatant was carefully discarded, leaving 25 μL of pellet remaining in each tube. Ultrapure water was then added to resuspend the pellets: 1960 μL to tubes I and II, and 0.565 mL to tube III. The entire contents of all three tubes were combined into a 20 mL scintillation vial, yielding a total of 1.69 mL of resuspended GNU. Finally, 0.35 mL of ultrapure water was added to the vial, resulting in a 2.035 mL 15 pM GNU solution.

### 2.4. Preparation of 3mM Hexanedithiol (HDT) Solution

An HDT solution was used as a molecular linker to immobilize GNU onto the PCB surface via gold–thiol bonding. To prepare the solution, 1 µL of HDT was mixed with 1999 µL of reagent-grade ethanol in a 20 mL glass scintillation vial, which was immediately sealed. The mixture was vortexed at 1800 rpm to produce 2 mL of 3 mM ethanolic HDT solution.

### 2.5. Preparation of 1 μM and 10 mM R6G Solutions

To prepare a 10 mM R6G solution, 7.1 mg of R6G was weighed out in the fume hood on an analytical balance and transferred to a 2 mL microcentrifuge tube. Then, 1.5 mL of distilled water was added to the tube to reach a 10 mM concentration. Afterwards, 1 μL of this prepared solution was then diluted in 10 mL of distilled water to prepare the 1 μM solution.

### 2.6. Adsorption of R6G Solutions on Substrates

10 μL of the 1 μM R6G solution was carefully added to both active areas of the functionalized PCB labelled “GNU”, and incubated for 20 min. Then, 10 μL of the 1 mM R6G solution was carefully added to both active areas of the unlabelled PCB and incubated for 20 min. Both substrates were characterized using FTIR spectroscopy.

### 2.7. Preparation of 5 mM PEG Solution

50 mg of 5 k HS-PEG-COOH was weighed on an analytical balance, then transferred to a 20 mL scintillation vial with 2 mL ultrapure water. The vial was vortexed at 1800 rpm for 30 s to dissolve the PEG powder. The solution was stored in a fridge until further use.

### 2.8. Functionalization of PCB with HDT and GNU

The concentrated GNU was immobilized on a gold-plated PCB surface via gold–thiol bonds. Briefly, the PCB substrate was immersed in a 50 mL beaker containing 2 mL of prepared 3 mM ethanolic HDT solution and sonicated at 60 kHz and 60% power for 2 min to allow gold–thiol bonds to form between the gold surface and HDT molecules. After sonication, the substrate was rinsed thoroughly with 70% ethanol, followed by ultrapure water inside a biosafety cabinet. The functionalized substrate was then incubated in 800 μL of the previously prepared 15 pM GNU solution for 10 min at 150 rpm, allowing GNU particles to bind to the free thiol groups of the HDT layer. Following incubation, the substrate was rinsed thoroughly with ultrapure water and air-dried in the biosafety cabinet. The functionalized PCB was characterized using FTIR spectroscopy.

### 2.9. PEG Functionalization of GNU-HDT

The GNU-HDT was further functionalized with thiol- and -COOH-terminated PEG linkers. The GNU-HDT substrate was placed in a beaker containing 800 μL of 5 mM PEG solution. The beaker was capped and incubated overnight on an orbital shaker at 150 rpm and room temperature. The following morning, the substrate was rinsed thoroughly with ultrapure water to remove any unbound PEG, then air-dried.

### 2.10. Preparation and Functionalization of PEG with ADH

Within the biosafety cabinet, 58.2 mg of ADH and 29.1 mg of EDC were weighed out and dissolved in 2 mL of ultrapure water in a scintillation vial. The solution was vortexed at 1800 rpm, resulting in a 167 mM ADH and 76 mM EDC solution. A PEG-functionalized PCB was placed in a clean 50 mL beaker, and 800 μL of the prepared ADH/EDC solution was added. The beaker was capped and placed on an orbital shaker at 150 rpm for 3 h. After incubation, the PCB was rinsed thoroughly with ultrapure water to remove unbound reagents, then left to air dry.

### 2.11. Antibody Buffer Exchange

A PBS to sodium phosphate buffer exchange was performed for the antibodies. Within a biosafety cabinet, 14.19 mg of sodium phosphate monobasic (NaH_2_PO_4_) was weighed out and dissolved in 1 mL of ultrapure water to prepare a 100 mM buffer solution. A 25 μL aliquot of 1 mg/mL HER-II and CA 15-3 mAb, originally supplied in PBS buffer, was thawed and transferred into a 30 kDa molecular weight cutoff centrifugal filter to exchange for 25 µL of 100 mM sodium phosphate. A second centrifugal filter was prepared containing 25 μL of ultrapure water for balance. The filters were centrifuged at 14,000 rpm for 10 min. After centrifugation, the filtrate containing the PBS was discarded. 25 μL of the prepared 100 mM NaH_2_PO_4_ buffer was added to a clean microcentrifuge tube to recover the mAb in the new buffer. The filter unit containing the concentrated mAb was inverted and placed into the recovery tube, followed by a reverse spin at 3000 rpm for 2 min. The filter unit was disposed of after centrifugation, and the microcentrifuge tube now contained 25 μL of HER-II and CA 15-3 mAb at 1 mg/mL in sodium phosphate buffer.

### 2.12. Antibody Preparation

A 0.15 M sodium chloride (NaCl), 10 mM sodium phosphate (NaH_2_PO_4_) buffer at pH 7.5 was prepared by dissolving 87.68 mg of NaCl and 14.02 mg of NaH_2_PO_4_ in 6 mL of ultrapure water in a 20 mL scintillation vial. The pH was lowered to 7.5 using hydrochloric acid while monitoring with a calibrated pH meter. Once the appropriate pH was achieved, the solution was transferred to a 10 mL graduated cylinder and diluted to volume with ultrapure water. The final buffer was stored in a fridge at 4 °C until use. Following buffer preparation, the mAb were activated using sodium periodate (NaIO_4_). To prepare this solution, 2.14 mg of NaIO_4_ was dissolved in 100 μL of ultrapure water in a clean microcentrifuge tube, vortexed, and covered with aluminum foil to protect it from light. Then, 5 μL of this freshly prepared NaIO_4_ solution was added to 25 μL of mAb. The mixture was vortexed at 2200 rpm for 30 s and incubated on an orbital shaker at 275 rpm for 45 min. To quench the oxidation reaction, 200 μL of 1× PBS was immediately added. Finally, the original antibody buffer (~230 μL) was exchanged with 150 μL of the prepared coupling buffer using centrifugal filter units, following previously described protocols. The coupling buffer was a mixture of 10 mM (NaH_2_PO_4_), and 0.15 mM (NaCl) dissolved in ultrapure water pH 7.5.

### 2.13. Antibody Conjugation

After completing the 3 h ADH/EDC incubation, 10 μL of the prepared CA mAb was added to the first active area and 10 μL of the HER-II mAb to the second active area on the PCB channel. The PCB substrate was then carefully placed in an airtight container to minimize evaporation and incubated overnight at room temperature on an orbital shaker set to 150 rpm, allowing the antibodies to bind to the substrate. The next morning, under sterile conditions in a biosafety cabinet, 1 μL of 1 M ethanolamine was added to each active area to stabilize the HDT linkage and prevent reversal of the ADH-antibody bond. This reaction proceeded for 1 h at room temperature on the shaker at 150 rpm. To block unreacted sites, a 10% (*w*/*v*) BSA solution was prepared by dissolving 6.0 mg BSA in 60 μL of coupling buffer. Then, 15 μL of this blocking solution was applied to the surface of the active areas and incubated for 10 min at room temperature on the orbital shaker at 150 rpm. Following incubation, the surface was rinsed thoroughly with 1× PBS to remove excess BSA. At this point, antibody conjugation to the nanostructured PCB substrate was complete.

It is important to note that in random (mAb) orientations, a higher number of mAbs may be immobilized; however, the accessibility of the antigen-binding (Fab) regions is often reduced. In contrast, oriented immobilization typically results in fewer mAb being loaded, but with greater exposure and accessibility of the antigen-binding sites. In random orientations—commonly achieved through conventional EDC/NHS coupling—mAbs can be conjugated via any available primary amine group, which is present in both constant Fc and variable Fab regions. This non-selective attachment can result in conjugation near or at the antigen-binding site, potentially hindering target recognition and decreasing detection sensitivity. In contrast, oriented immobilization-proposed here using EDC/ADH chemistry-targets glycosylation sites in the Fc region, forming stable hydrazone bonds that anchor the antibody to the GNU at the Fc end. This directional attachment preserves the functionality of the Fab regions, allowing more effective biomarker interaction despite a lower overall antibody density.

### 2.14. Microfluidic Chip Fabrication

The substrate was cleaned by rinsing with 70% ethanol (BP82031GAL, Fisher Scientific, Ottawa, ON, Canada) to remove any particulates. It was placed in a small circular container, where 70% ethanol was introduced into the channel and left for 30 s before being removed with a Kimwipe. The sample was then allowed to air dry. A 1.7 × 3.5 cm PDMS sheet was cut, and two holes were punched for the microfluidic ports using a 16-guage blunt needle. The prepared PDMS sheet was attached to the PCB using LePage 100% Repair Gel. After the adhesive had fully dried, two microfluidic couplers were installed on the substrate to serve as inlet and outlet ports. The PCB and PDMS were secured together using two binder clips until the adhesive set. After 30 min, the clips were removed, and PBS was carefully injected into the channel using a 16-gauge needle connected to a 1 mL syringe to check for leaks.

### 2.15. Microfluidic Setup and Interaction

**Figure 1:**
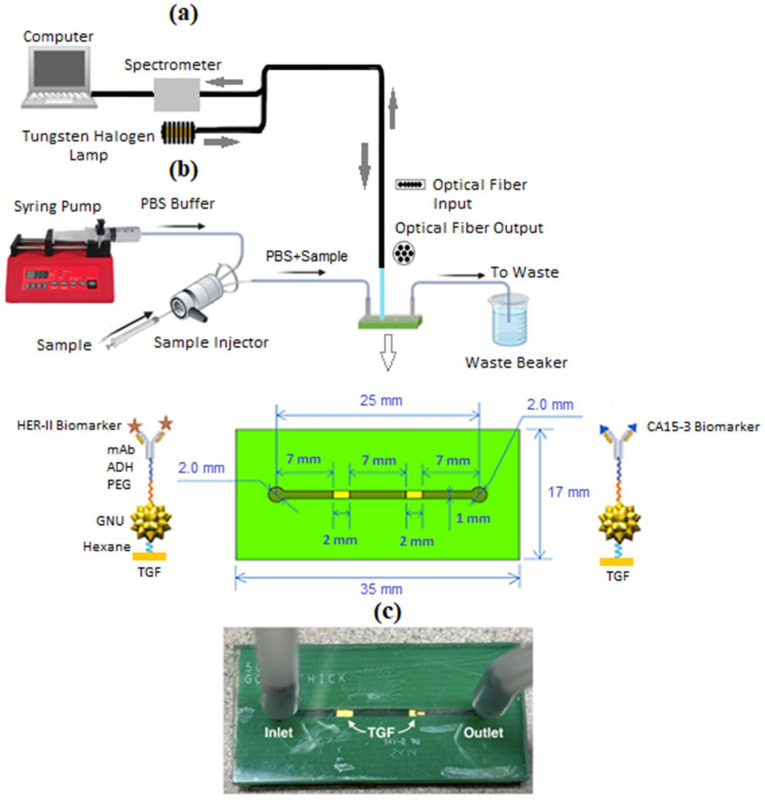
(**a**) Schematic of the experimental setup for the SE-FTIR spectroscopy, where the light source illuminates the active regions after the biomarker interaction phase. (**b**) The microfluidic system comprises a syringe pump, sample injector, and microfluidic substrate connected via tubing. (**c**) GNU-immobilized TGFs conjugated directionally with HER-II and CA15 mAbs via ADH.

Figure 1 illustrates the experimental setup. Figure 1a illustrates the FT-NIR setup in reflectance mode, where the active sensing zones were analyzed using an FT-NIR Nano Quest spectrometer (NanoQuest 2.5, Ocean Insight, Orlando, FL, USA). The spectrometer’s integration time was set to 5 s, at 8 nm resolution. A 200 µm bifurcated reflectance probe (RP28, Thorlabs, Newton, NJ, USA) was used, with the source leg connected to a tungsten halogen lamp (HL-2000-HP, Ocean Insight), delivering light to the sample at a distance 10 mm perpendicular to the substrate. The collection leg delivered the reflected light to the spectrometer for analysis. The background reading was a solid white foam surface. In Figure 1b, the microfluidic platform comprises a syringe pump, a sample injector including the needle port and sample loop configuration, and a microfluidic substrate connected via tubing. The halogen light source illuminates two distinct GNU-immobilized TGFs modified with HER-II and CA15-3 mAbs directionally conjugated via ADH chemistry for optimal orientation and antigen binding. Flow optimization was performed using dye. Sample injection to channel exit took 1 h 10 min, with 43.15 min from inlet to outlet. The system was primed with 1X PBS (pH 6) at 10 μL/min for 5 min. Then, 4 μL of BCS sample was diluted in 56 mL of PBS, and 140 μL of 0.1 M of MES buffer was injected at 1 μL/min for 1 h 15 min. post-interaction, SE-FTIR spectra were recorded at active zones and analyzed using SV, a custom Python 3.12-based tool.

## 3. Quantification

### 3.1. Calibration

Two different methods were explored to identify and quantify BCS data points along the calibration curve: (a) using a single wavenumber associated with the most prominent or consistently overlapping SE-FTIR peak, and (b) detecting the most frequently observed peaks within a specified spectral window. The first method encountered challenges due to variations in spectral profiles and peak shifting between samples. As a result, fixing the analysis to one wavenumber often missed the actual peak location, leading to less accurate data interpretation. In comparison, the second method proved to be more reliable, offering improved consistency and a stronger correlation between spectral intensity and analyte concentration.

To improve quantification and account for slight spectral variations, this method focused on identifying the most consistently observed peaks within a selected spectral window. A refined peak-detection algorithm was applied, configured to capture only the most significant FTIR peaks. The intensities of these peaks were then mapped to analyte concentrations using a pre-established calibration curve. The process began with visualizing the raw spectral data in stacked plots to highlight recurring peak patterns. Following this, the spectra were normalized using the kin/max normalization technique and re-plotted for comparison. Both raw and normalized FTIR spectra for each biomarker were then analyzed using an automated algorithm that determined the optimal wavenumber range-based on position and width (ranging from 20 to 370 wavenumbers), which exhibited the strongest inverse linear trend between concentration and peak reflectance. The quality of this relationship was measured by the R^2^ value of the corresponding linear regression. All samples were included in the analysis without exclusion.

### 3.2. Development of Spectra View (SV) Software

Preprocessing plays a critical role in enhancing FTIR spectral quality by reducing noise and ensuring accurate quantitative analysis. Essential steps include signal filtering, baseline correction (such as fluorescence removal), and normalization to correct for factors like laser intensity fluctuations. In this study, spectral data were processed using SV, a custom Python-based tool developed for Raman data visualization and concentration estimation. SV performs two primary functions: (1) visualizing FTIR spectra, and (2) estimating sample concentrations by analyzing key spectral features, particularly within the 6700–6800 cm^−1^ and 6480–6580 cm^−1^ regions, respectively. The processing pipeline includes spectral smoothing using the *filtfilt* function from the *scipy.signal* library and baseline correction via the *isals* method from the *pybaselines.Whittaker* module. After these preprocessing steps, a peak detection algorithm identifies the most prominent peaks within the defined spectral window. The resulting peak intensities are fed into a linear regression model, which estimates analyte concentrations based on calibration curves generated for each pure biomarker. To increase robustness, SV evaluates a spectral region rather than relying on a fixed wavenumber, minimizing errors caused by peak shifting. The tool is compatible with Ocean Optics spectrometers and integrates seamlessly with their software to facilitate data import. The current model delivers optimal performance within the 10–100 ng/mL (or U/mL) concentration range.

## 4. Characterization

### 4.1. UV-Vis Absorbance

**Figure 2:**
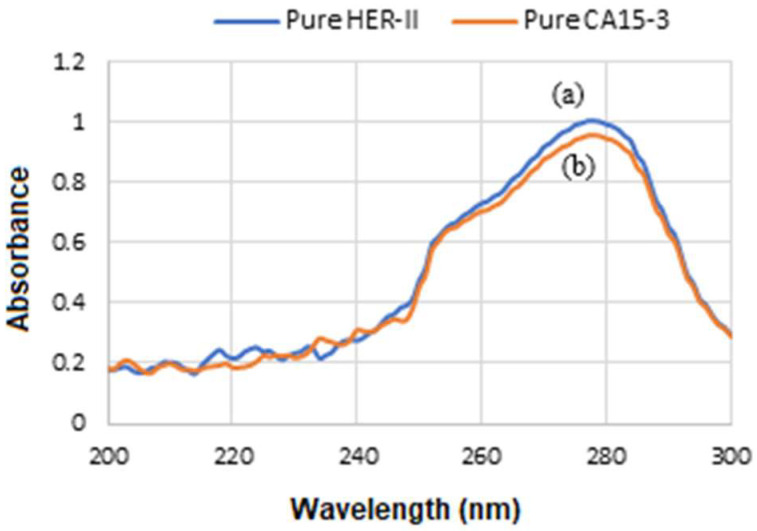
(**a**) UV-Vis absorbance of standard HER-II and (**b**) CA15-3 biomarkers.

UV-Vis analysis of GNU functionalization was conducted using a Jenway 7205 spectrophotometer, covering a wavelength range of 198 to 800 nm. Since HER-II is a large transmembrane glycoprotein, its UV-Vis absorbance spectrum is not sharply defined like dyes or organic molecules. The pure HER-II absorbs UV light strongly at about 280 nm due to its protein backbone and aromatic amino acid content, as seen in Figure 2a, which is due to the aromatic amino acids, especially tryptophan (Trp), tyrosine (Tyr), and phenylalanine (Phe) in the protein structure. The peak about 280 nm primarily corresponds to Trp residues, which are generally structural and contribute to the proper folding and stability of the protein and are used to quantify HER-II in purified samples. Figure 2b shows that the standard CA 15-3 exhibits slightly lower absorbance peak around 280 nm than HER-II, which is primarily due to the protein portion of MUC1, particularly the aromatic amino acids Trp and Tyr. For both HER-II and CA 15-3, a UV-Vis absorption peak near 280 nm confirms the presence of their protein components, despite carbohydrate modifications. Since carbohydrates absorb minimally at this wavelength, the 280 nm signal reflects only the protein content. This distinction is crucial when analyzing heavily glycosylated biomarkers such as CA 15-3. Cancer Antigen 15-3 is a glycoprotein linked to tumours and is commonly utilized as a biomarker for tracking the progression of breast cancer. In cancer patients, elevated levels of specific proteins—particularly those involved in inflammation and immune response—are often observed. These changes indicate alterations in the overall serum protein profile, with certain proteins increasing or decreasing in abundance.

### 4.2. Enhancement Factor (EF)

A 25 μL aliquot of the 10 mM R6G solution was gently applied onto a glass slide to prepare the sample, which was then analyzed using FTIR spectroscopy. To evaluate the sensor’s enhancement factor (*EF*), 10 μL of R6G at concentrations of 10 mM and 1 μM were introduced as Raman-active probes onto both PCB-TFG and PANTF substrates. For the PANTF sample, the 10 μL volume accounted for the larger surface area of the GNU during SE-FTIR measurements. The observed enhancement is attributed to electrostatic interactions between the positively charged R6G molecules and the inherently negative surface charge of the GNU. Additionally, fluctuations in signal intensity are believed to result from various charge transfer mechanisms, likely caused by dynamic changes in the molecular environment associated with the random movement of R6G molecules on the metallic surface during the adsorption process [33,34]. The enhancement factor at a specific power level was calculated based on the intensity of lines amplified by localized surface plasmon resonance (LSPR), using Equation (1) [35].

(1) $$EF=\frac{I_{SE-FTIR}}{I_{FTIR}}\times \frac{C_{FTIR}}{C_{SE-FTIR}}$$

where $I_{FTIR}$ is the intensity of the PCB-TGF substrate with R6G at a concentration $C_{FTIR}$ = 10 mM, $I_{SE-FTIR}$ is the intensity of the GNU-immobilized substrate with R6G at a concentration $C_{SE-FTIR}$ = 1 μM, so $\frac{C_{FTIR}}{C_{SE-FTIR}}$ = 10^4^.

**Figure 3:**
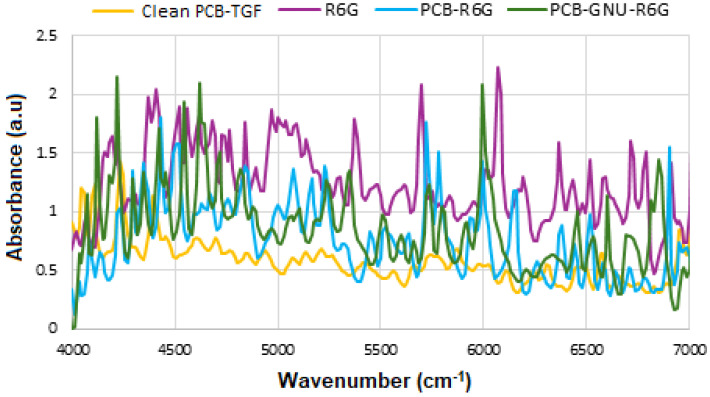
Absorbance FTIR spectra of PCB, R6G, PCB-R6G, and PCB-GNU-R6G, respectively.

When comparing the R6G, PCB-R6G and PCB-GNU-R6G absorbance spectra in Figure 3, there were four regions where peaks from all three spectra are overlapped, albeit slightly shifted. Those regions were ~5300 cm^−1^, ~5700 cm^−1^ and ~6000 and ~6700 cm^−1^. These peaks may be tentatively attributed to R6G, and be used for the enhancement factor calculations. The results are summarized in Table 2.

### 4.3. Flow Optimization

**Figure 4:**
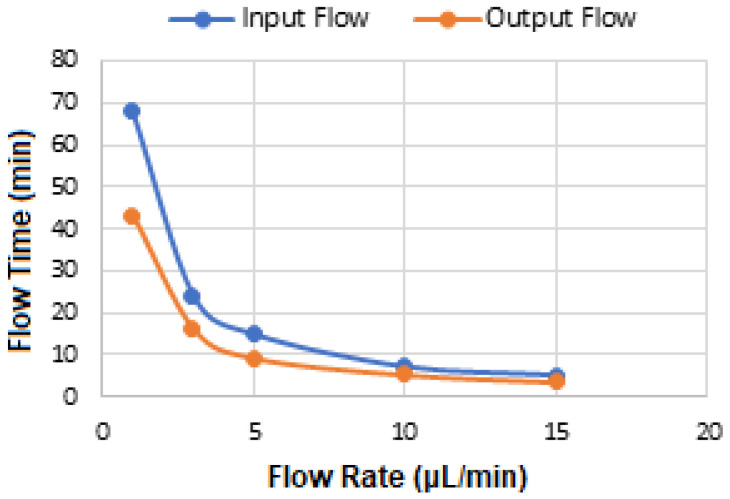
Variation in Flow time versus flow rate of dye in a microfluidic channel.

Figure 4 shows the optimization of flow time versus the flow rate using dye. It took about 70 min for the sample, after injection, to exit the channel (outflow), out of which about 43 min was spent travelling the entire surface of the channel within the substrate from the inlet to the outlet ports. Therefore, 4 μL of the BCS sample was diluted in 56 mL of PBS, and 140 μL of 0.1 M MES buffer was injected at 1 μL/min for 1 h 15 min.

## 5. Results and Discussion

The unique spiky core-tip structures significantly enhance the efficiency of biosensing and therapeutic applications by providing a multitude of plasmonic hotspots and enabling a higher degree of surface functionalization with targeting molecules [36]. These nanoscale features increase the active surface area, enabling the attachment of a diverse array of targeting agents, including mAbs, peptides, cell surface ligands, and aptamers. Through this targeted surface functionalization and conjugation, these nanostructures serve as highly effective label-free Surface-Enhanced Raman Scattering (SERS) and Surface-Enhanced Fourier Transform (SE-FTIR) probes. Such probes offer a promising alternative technology for rapid, ultrasensitive detection of low-abundance biomarkers, which is critical for improving the sensitivity and specificity of cancer diagnostics and early disease detection [23,24,37,38,39,40]. To maximize the performance of immunosensors, particularly in terms of capacity, stability, and reproducibility, antibodies are typically immobilized onto solid substrates. This immobilization not only enhances the long-term stability of the biorecognition elements but also ensures consistent sensor performance over repeated use. Together, the combination of advanced nanostructure design and robust biofunctionalization strategies is driving forward the development of next-generation biosensors with improved diagnostic capabilities.

### 5.1. SE-FTIR of Standard HER-II

**Figure 5:**
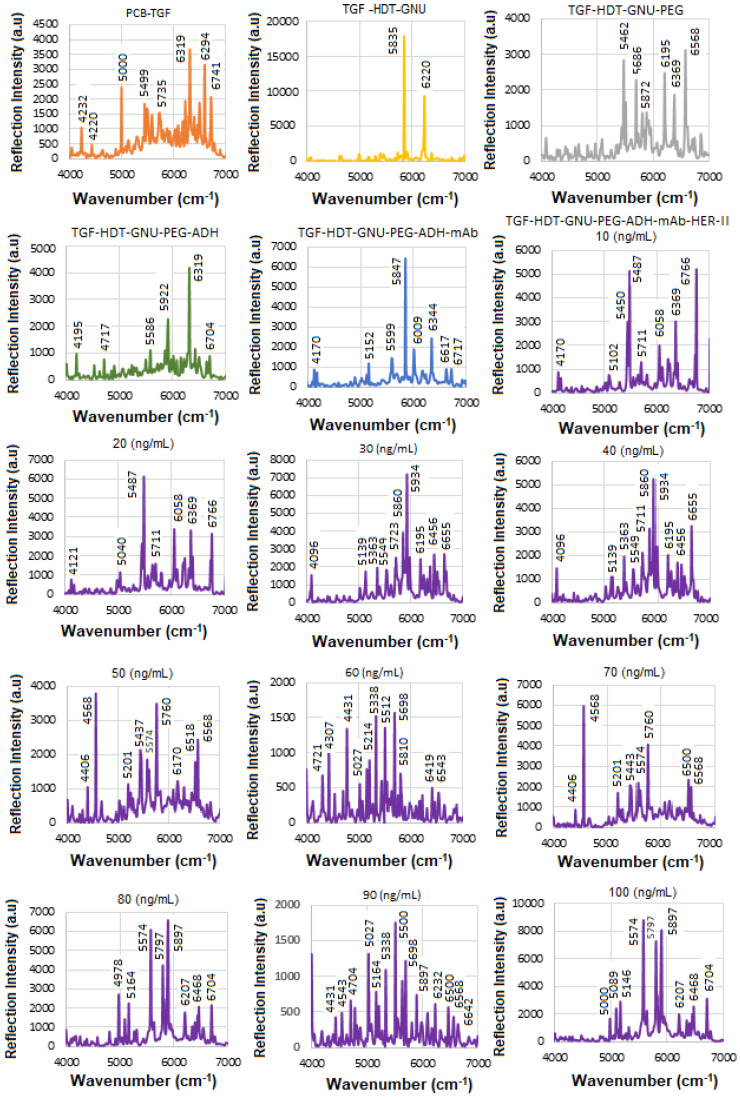
The trials of the SE-FTIR results for functionalization, conjugation, and interaction steps of the standard HER-II biomarker.

The solid substrate provides a stable platform for precise surface chemistry, and it is compatible with label-free detection, i.e., no need for fluorescent tags or reporters. The next step was to perform SE-FTIR spectroscopy using standard HER-II samples with known concentrations to generate a calibration curve. This curve was later used to quantify the concentration of a real sample with an unknown value. While the total protein level in healthy serum typically remains within a normal range, cancer can alter the types and concentrations of specific proteins. Rather than causing a general increase in total protein, cancer often leads to elevated levels of certain proteins, particularly those related to inflammation and immune responses. These changes reflect disruptions in normal cellular processes and can shift the overall protein profile in the serum, with some proteins becoming more or less abundant. Figure 5 presents the NIR-FT line intensities for various samples during functionalization, conjugation, and interaction stages in the 4000–7000 cm^−1^ range, where each interaction was repeated three times, and the average values were used to construct the calibration curve. Based on the superposition principle, the ideal (noise-free) model function representing the protein FTIR spectrum at a given wavenumber and temperature is defined as follows [41].

(2) $$\gamma (\bar{v},T)=\sum_{k^{\prime }=1}^{N}c_{k^{\prime }}(T)\varepsilon_{k^{\prime }}(\bar{v})$$

The summation is performed over N absorption bands that are characteristic of specific secondary structure elements or amino acid side chains, and are assumed to be independent of temperature. The shape of each band $k^{\prime }$ is defined by a specific function $\varepsilon_{k}(\bar{v})$, while the amplitude of this function $c_{k}$ may potentially vary with temperature. The relationships between these parameters enable the analysis of FT-NIR peaks, focusing on both the peak position and the importance of the peak amplitude.

During the interaction, infrared (IR) light passes through the sample and reflects off the substrate, offering valuable insights into the material’s molecular structure and bonding. The sample must be sufficiently thin to allow IR transmission without complete absorption. Initially, with only TGF present, most reflections occur between. The results for TGF_50_-immobilized GNUs via HDT show the features of hexane (n-hexane) in the 4000–7000 cm^−1^ range corresponding to overtone and combination bands rather than fundamental vibrations. There are two prominent overtone peaks of its methyl (–CH_3_) groups in this region, one around 5907 cm^−1^ and another near 5870 cm^−1^ (in our case, 5835 cm^−1^), respectively, both of which are part of the first overtone of the C–H stretching region.

The orientation of PEG molecules on the GNU surface can be evaluated before mAb conjugation by analyzing detection sensitivity related to their binding configuration. “Top bonding” corresponds to interactions at the COOH terminus of the PEG chain, while “bottom bonding” refers to C–S and S–Au bonds formed close to the GNU surface. Given PEG’s simpler structure compared to mAbs, its in-plane and out-of-plane vibrational modes can provide useful information about whether the molecules are aligned parallel or perpendicular to the surface. In this work, PEG attachment is achieved through ADH-mediated linkage. The PEG-related peaks are divided into weak (4000–5000 cm^−1^) and strong regions (5000–7000 cm^−1^), respectively. The 4000–4650 cm^−1^ region corresponds to C–H and CH_2_ combination bands. Features between 4545 and 5333 cm^−1^ are associated with O–H combination and overtone bands. The 5556–6061 cm^−1^ range reflects C–H and CH_2_ first overtones, while the 6000–7000 cm^−1^ region contains a broad envelope of overlapping O–H, C–H, and CH_2_ overtone and combination bands.

The FTIR spectrum of ADH is characterized by functional groups typical of: Hydrazide groups (CO–NH–NH_2_), amide linkages, NH_2_ and NH stretching and bending, C=O (carbonyl) stretching, and aliphatic CH_2_ chain from the adipic acid backbone. They typically exhibit overtone and combination bands within the NIR region including: (a) C–H Combination Bands (4000–4650 cm^−1^) where multiple methylene (–CH_2_–) stretches and bends would likely contribute to combination bands here, similar to other aliphatic compounds, (b) amide/N–H overtone and combination bands (4500–5500 cm^−1^) where hydrazide N–H stretching combinations and overtone bands could appear in this intermediate range, and (c) C–H first overtones (5500–6000 cm^−1^) where the overtones from C–H stretching in methylene units would be expected here. The intermediate range corresponding to the CH_2_ band between 5800 and 6000 cm^−1^ is attributed to 2nd overtone of C–H stretch, which is near the 6500–6800 cm^−1^ range of N–H first overtones, though more commonly beyond 6000 cm^−1^, and combination bands involving N–H + C=O or N–H + CH_2_ modes.

mAbs are highly glycosylated proteins and are made up of largely amide vibrations from the peptide bonds present within the protein structure. Therefore, the spectra often reflect both the protein backbone (amide groups, aromatic residues) and carbohydrate moieties (polysaccharide-linked sugars, OH groups). The secondary structure elements, such as α-helices vs. β-sheets, can be inferred via hallmark overtone frequencies like ~5755 cm^−1^ (helix) and ~5915 cm^−1^ (sheet). One class of globular proteins is the γ-globulins, which include immunoglobulins (such as IgG), commonly known as antibodies. These proteins are a subclass of γ-globulins and are primarily composed of 60–70% β-sheet structures, with minimal α-helix content [42,43]. Formulation components such as sugars (e.g., glucose, sucrose) may introduce significant OH-related overtone peaks, often overlapping with protein bands. In the 4000–7000 cm^−1^ NIR region, mAbs typically exhibit a complex profile comprising: CH_2_ and CH combination/overtone bands (4000–4500 cm^−1^), amide-related combinations (~4700–4900 cm^−1^), overtones from OH groups (sugars or glycosylation) (~5800–6000 cm^−1^), aromatic CH overtones and first overtone of OH (glucose) (~6060–6210 cm^−1^), overtones indicating protein secondary structure (~5755 and ~5915 cm^−1^), OH first overtone from glucose/carbohydrates, first overtone of N-H, RNH_2_ groups from protein and peptide amines due to polypeptides and antibody contribution (~6600–6800 cm^−1^), stronger O–H first overtone components, possibly more of the N–H first overtone, and overlapping broad combinations from CH groups (~6800–7000 cm^−1^). The results after conjugation of GNU-PEG with the mAb, comprised mostly amide vibrations from the peptide bonds within the structure of the protein.

Table 3 summarizes the FTIR results for HER-II biomarker illustrated in Figure 4.

**Figure 6:**
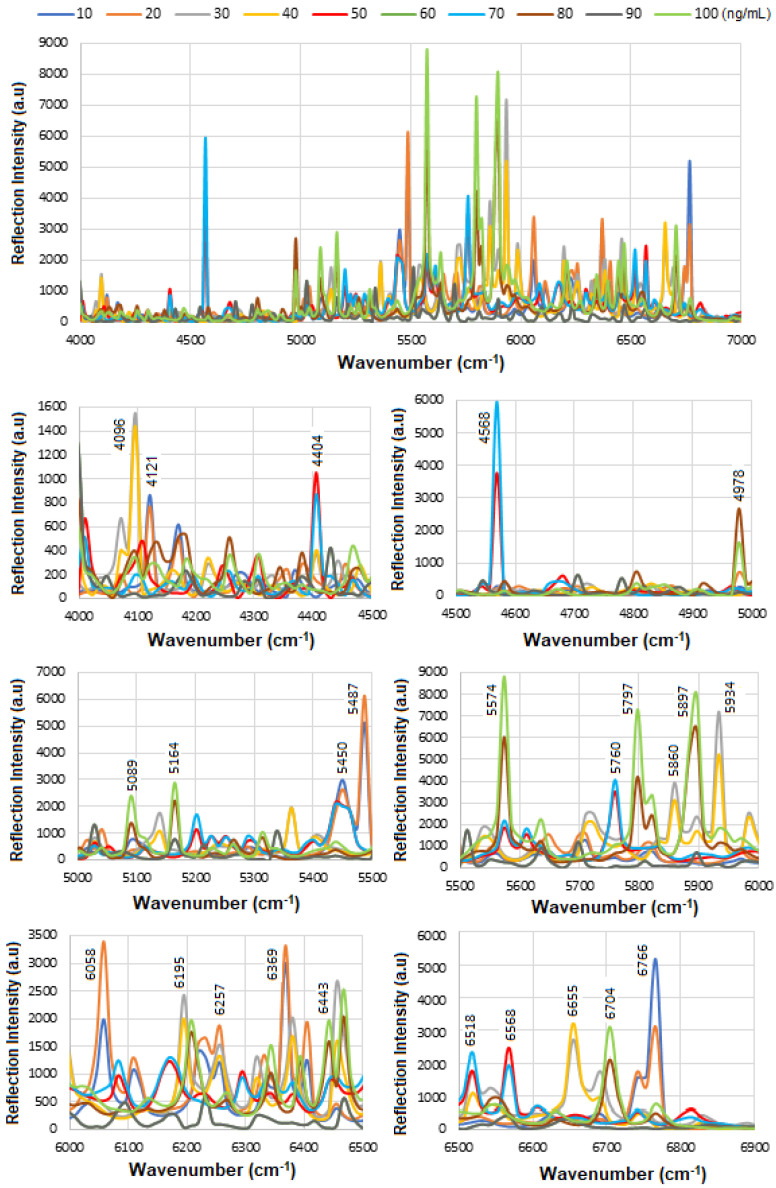
Overlapped variation in pure HER-II reflection intensity versus the wavenumber.

Figure 6 presents the overlapped spectra of all concentrations for standard HER-II, segmented into defined regions to delineate the areas with the highest degree of spectral overlap. In SE-FTIR enhancement, the local EM fields at the surface (near the molecule) are stronger due to plasmonic resonance or interference. This stronger field enhances the molecule’s ability to interact with IR light. A reflective surface is used to measure small absorption-induced changes in reflectivity. The enhancement comes from increased local EM fields, not from having higher reflectivity itself. What matters is the change in reflectance due to absorption, not the absolute reflectivity. In general, higher reflectivity in a spectrum implies lower apparent absorption, which could indicate a lower concentration of a biomarker. The vibrational absorption by the biomarker causes a decrease in the reflectance spectrum at specific wavenumbers. A stronger absorption (due to higher concentration or stronger transition dipole moment) produces a stronger decrease, which implies a lower reflectance at that frequency. Similarly, a weaker absorption (lower concentration) results in a lower decrease, i.e., higher reflectance at that frequency. Therefore, higher reflectance at a biomarker’s absorption frequency generally implies lower absorption, which in turn suggests lower biomarker concentration. It is noteworthy that biological samples (like serum, tissue, etc.) have complex spectral backgrounds where one is interested in tracking specific vibrational bands (e.g., amide I, phosphates, lipids). A reduction in the depth of these absorption bands (higher reflectance) can indicate a drop in concentration, useful for early detection or monitoring. However, reliable quantification requires good calibration, control of background signals, and the use of multivariate analysis (e.g., PCA, PLS) for complex biosamples.

**Figure 7:**
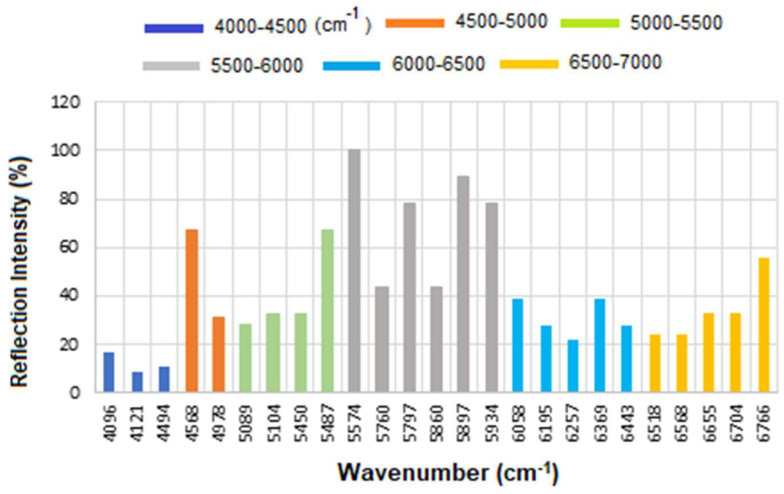
Histogram illustrating the overlapping SE-FTIR spectra of standard HER-II within the spectral range of 4000–7000 cm^−1^.

The corresponding results are presented as a histogram in Figure 7 to enhance the visualization of regions with varying reflectance. The region between 4000 and 4500 cm^−1^ has the lowest reflectance, i.e., highest absorption, mainly related to CH_2_ and CH combination/overtone bands. The region from 6000 to 7000 cm^−1^ represents the next highest absorption band, associated with overtones indicative of protein secondary structures, RNH_2_ and OH first overtone features, and broad combination bands from CH groups.

**Figure 8:**
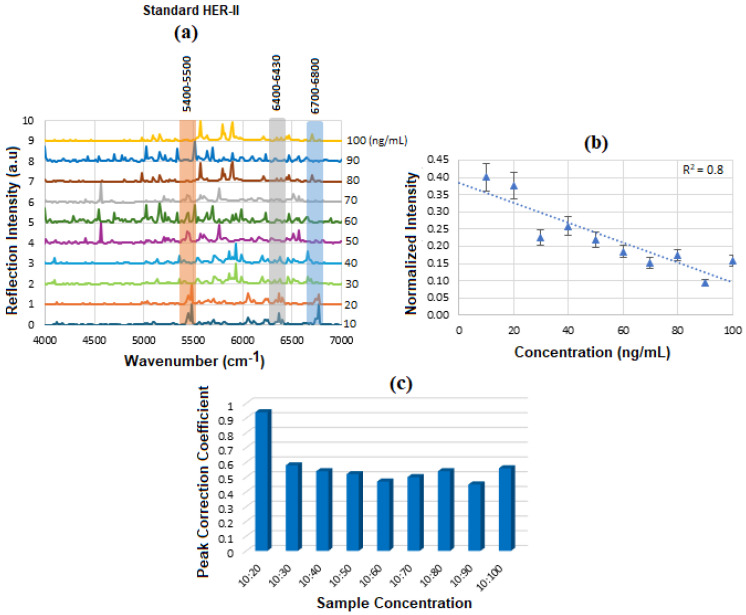
(**a**) Corresponding SE-FTIR spectra for standard HER-II solution at various concentrations where the intensity decreases with concentration, (**b**) Calibration curve for standard HER-II biomarker based on the average value in Figure 4, with a sensitivity limit of about 10 ng/mL, the SE-FTIR intensity varies linearly with concentration, and (**c**) Cross-correlation of the standard HER-II biomarker concentrations.

Figure 8a displays the stacked SE-FTIR spectra of standard HER-II solutions at concentrations ranging from 10 to 100 ng/mL. The strip regions between 5400 and 5500, 6400 and 6430, and 6700 and 6800 cm^−1^ are likely related to the overtone or combinations, OH first stretch overtones (especially of glucose), aromatic CH first overtone, and CH_2_ combination overtones. These values are used to construct the calibration curve. As the HER-II concentration increases, the intensity of reflection decreases linearly, making this region particularly suitable for quantitative analysis. Figure 8b presents the calibration curve, generated by plotting the SE-FTIR signal intensities of the characteristic RNH_2_ and OH first overtone features against their corresponding HER-II concentrations. The slopes of the curves for each region were found to be 0.11, 0.34, and 0.80, respectively. Among these, the slope of R^2^ = 0.80 was selected as the optimal value, representing the sensitivity of the SE-FTIR immunoassay. It is important to note that while individual FTIR spectra reveal the molecular composition at specific sample points, they may not reflect the broader sample due to the lack of spectral averaging. Nonetheless, the consistent linear decrease in reflection signal intensity across concentrations confirms the sensor substrate’s uniformity and tentative, reliable response behaviour throughout the experiment.

FCC is a statistical tool used in signal processing and data analysis to measure similarity between two signals as a function of time or spatial lag. It helps identify shared patterns between signals.

When applied in the context of biomarker concentration, the FCC can help analyze spatial or signal-related patterns in relation to the biomarker’s distribution. The key parameter is the amplitude at time lag = 0, indicating signal similarity; a higher amplitude means higher similarity between the signals. Variations in FCC for a BC biomarker at different concentrations reflect changes in signal intensity, spectral features, or noise due to Experimental/Instrumental noise, Sample Preparation Variability, and Biological Variability. The above results are denoted as a histogram in Figure 8c, where the 10:20 exhibited the highest FCC, and the others had a lower correlation between 50 and 55%. The intensity value at the actual peak within a range of the SE-FTIR spectra was used to construct the calibration curve, which produced a better result than using a single maximum peak value.

### 5.2. SE-FTIR of Standard CA 15-3

**Figure 9:**
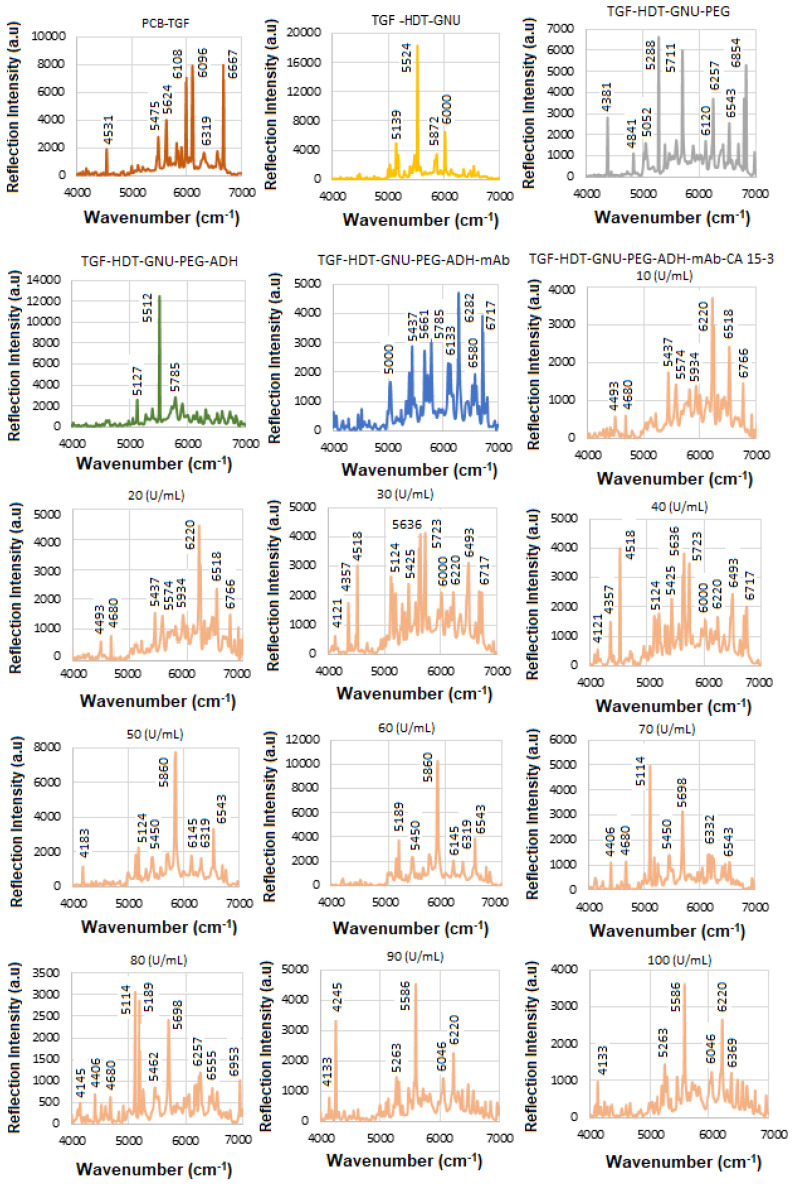
The trials of the SE-FTIR results for functionalization, conjugation, and interaction steps of the standard CA15-3 biomarker.

Figure 9 shows the results of the SE-FTIR for the standard CA15-3 biomarker using various concentrations. This biomarker is derived from MUC1 mucin, a large glycoprotein with a high molecular weight. In the range 4000–7000 cm^−1^, it is often considered as near-IR overtones or combination bands. The vibrational modes between ≈ 3200–3600 cm^−1^ are associated with O-H stretch and ≈3300 cm^−1^ to N-H stretch (amide A), between ≈ 2800–3000 cm^−1^ to C-H stretch (aliphatic). The overtone or combinations are expected to occur around 6600–7200 cm^−1,^ and the 1st overtone about 5800–6000 cm^−1^. The combination bands are somewhat above these values. There are some similarities between the proteins or glycoproteins, so both will show overtone/combination bands of N–H, O–H, C–H, etc. They have both overtones, including first overtones of O–H (around 7000–5500 cm^−1^), N–H, and possibly combination bands involving CH stretching/bending. Thus, there should be overlapping peaks in many of those regions. In both types of samples, water (free and bound) will contribute to those higher energy overtone regions, O–H overtones often interfere/overlap. At high enough concentration, both biomarkers will produce an overtone signal and overlapping peaks. However, some possible differences should be considered, such as glycosylation (more or different sugar moieties in CA15-3), and possibly different secondary structure content (α-helix vs. β-sheet) might shift relative intensities of overtones/combinations. Also, different levels of bound water and differences in the sample environment could alter O–H/N–H combination bands. Intensities might differ as CA15-3’s glycan parts may have more O–H from sugars, and HER-II (extracellular domain) may have a different hydrophobic/hydrophilic balance. They could have different shapes or widths of bands in ~6000–7000 cm^−1^ due to the degree of exposure of hydrophilic groups. Finally, the differences in concentration in biofluids may make one more detectable; as such, CA15-3 is often more abundant or used more in monitoring, while HER-II exodomain shedding may be at a lower concentration. Therefore, HER-II overtone bands might be weaker or noisier [44].

Table 4 shows the tentative CA15-3 FTIR peak assignments to the wavenumbers. These peaks are generally due to common functional groups found in glycoproteins, carbohydrates, and protein secondary structures, so they are also expected in CA15-3, which is a high-molecular-weight mucin-like glycoprotein.

**Figure 10:**
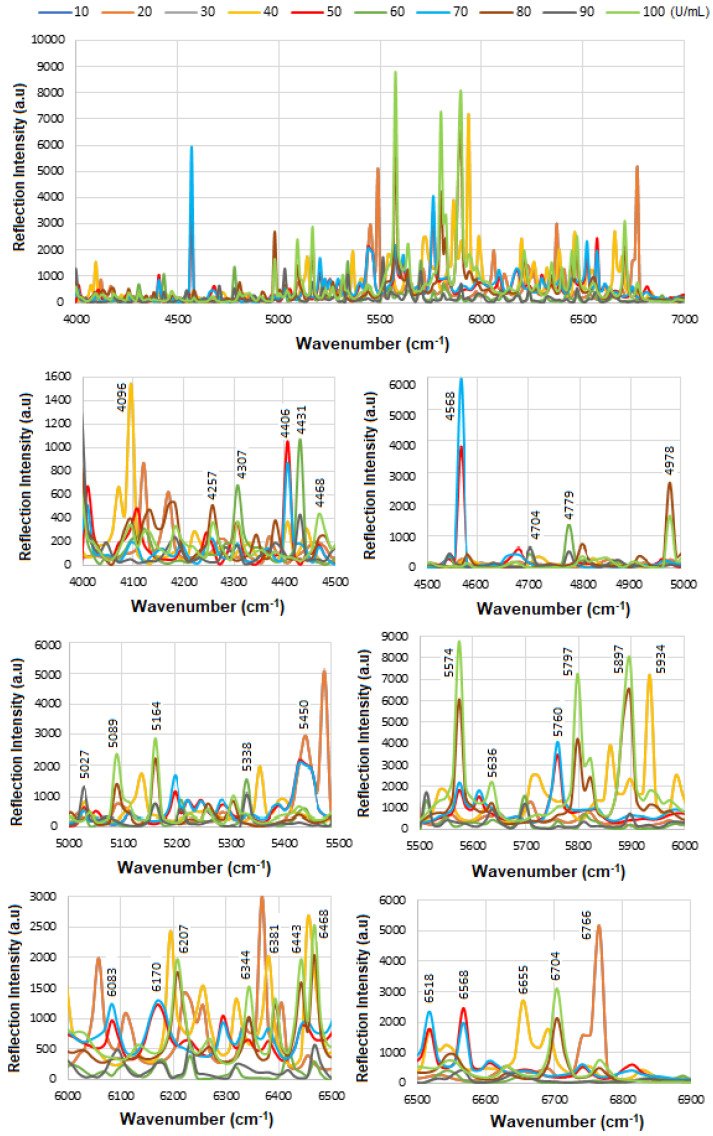
Overlapped variation in pure CA15-3 reflection intensity versus the wavenumber.

**Figure 11:**
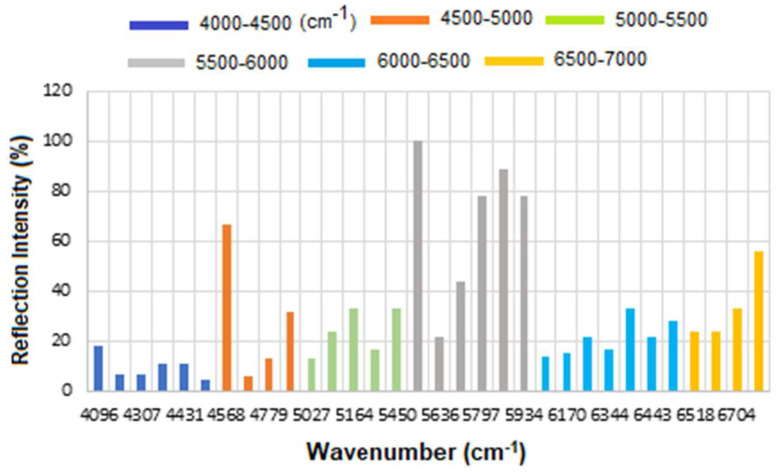
Histogram illustrating the overlapping SE-FTIR spectra of standard CA15-3 within the spectral range of 4000–7000 cm^−1^.

Therefore, while CA15-3 and HER-II share some general FT-NIR regions (due to similar biochemical components like proteins and carbohydrates), the specific peak positions and intensities will vary: Shared features: Broad overtones/combinations involving OH, CH, NH, RCOOH (glycoprotein features). The main differences are: HER-II has antibody-specific signals, whereas CA15-3 is dominated by mucin-type O-glycosylation and may lack sharp aromatic or antibody-based peaks. Figure 10 presents the overlapped spectra of all concentrations for standard CA15-3, segmented into defined regions to delineate the areas with the highest degree of spectral overlap. As explained above a stronger absorption due to higher concentration produces a stronger decrease, which means a lower reflectance at that frequency, and a weaker absorption (i.e., lower concentration) results in a lower decrease, i.e., higher reflectance at that frequency. The corresponding results are presented as a histogram in Figure 11. The region between 4000 and 4500 cm^−1^ has the lowest reflectance, similar to HER-II, i.e., highest absorption mainly related to CH_2_ and CH combination/overtone bands. The region from 6000 to 7000 cm^−1^ represents the next highest absorption band, associated with overtones indicative of protein secondary structures, RNH_2_ and OH first overtone features, and broad combination bands from CH groups. Comparison between HER-II (Figure 6) and CA15-3 (Figure 10) indicates: (a) Both biomarkers have lowest reflection between 400 and 4500 cm^−1^ with CA15-3 exhibiting more peaks, (b) Both biomarkers show a similar reflection between 4500 and 5000 cm^−1^, (c) CA15-3 show a lower reflection than HER-II between 5000 and 5500 cm^−1^, (d) Both biomarkers exhibit the highest reflection between 5500 and 6000 cm^−1^, (e) CA15-3 show a lower reflection tan HER-II between 6000 and 6500 cm^−1^, and (f) Both biomarkers show a similar reflection between 6500 and 7000 cm^−1^.

The corresponding results are illustrated as a histogram in Figure 11. The spectral region between 4000 and 4500 cm^−1^ exhibits the lowest reflectance, indicating the highest absorption—similar to that observed for HER-II. This absorption is primarily attributed to combination and overtone bands involving CH_2_ and CH groups. The 6000–7000 cm^−1^ range shows the next most significant absorption band, which corresponds to protein secondary structure overtones, as well as features related to RNH_2_, OH first overtones, and broad combination bands from CH-containing groups.

A comparative analysis of the reflectance spectra of HER-II (Figure 7) and CA15-3 (Figure 11) reveals the following: (a) Both biomarkers show the lowest reflectance (i.e., highest absorption) in the 4000–4500 cm^−1^ range, with CA15-3 displaying a greater number of distinct peaks, (b) Both biomarkers exhibit similar reflectance patterns between 4500 and 5000 cm^−1^, (c) In the 5000–5500 cm^−1^ region, CA15-3 shows lower reflectance compared to HER-II, (d) Both biomarkers demonstrate their highest reflectance in the 5500–6000 cm^−1^ range, (e) Between 6000 and 6500 cm^−1^, CA15-3 again shows lower reflectance than HER-II, and (f) In the 6500–7000 cm^−1^ region, the reflectance of both biomarkers is comparable.

**Figure 12:**
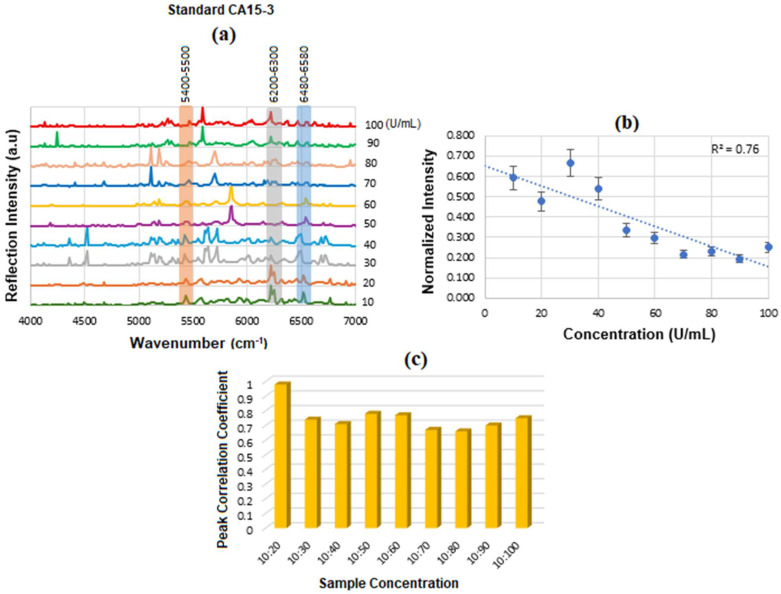
(**a**) SE-FTIR spectra of standard CA15-3 solutions at varying concentrations, showing a linear decrease in intensity within the 6480–6580 cm^−1^ region as concentration increases, (**b**) Calibration curve for the standard CA15-3 biomarker, based on the average values from Figure 4, demonstrating a linear relationship between SE-FTIR intensity and concentration, with a sensitivity limit of approximately 10 U/mL, and (**c**) Cross-correlation analysis of the standard CA15-3 biomarker concentrations.

Figure 12a displays the stacked SE-FTIR spectra of standard CA15-3 solutions at concentrations ranging from 10 to 100 U/mL. The strips between 5400 and 5500, 6200 and 6300, 6400 and 6500 cm^−1^ correspond to CH_2_/CH_3_ first stretch overtones (proteins/lipids) expected due to peptide backbone and lipid components, OH and CH overtones, which are common across many glycoproteins and present in CA15-3 too. As the CA15-3 concentration increases, the intensity of reflection decreases linearly, making this region particularly suitable for quantitative analysis. Figure 12b presents the calibration curve, generated by plotting the SE-FTIR signal intensities of the characteristic RNH_2_ and OH first overtone features against their corresponding CA15-3 concentrations. Similarly, the slopes of the curves for each region were found to be 0.40, 0.186, and 0.76, respectively. Among these, the slope of R^2^ = 0.76 was selected as the optimal value, representing the sensitivity of the SE-FTIR immunoassay. It is important to note that while individual FTIR spectra reveal the molecular composition at specific sample points, they may not reflect the broader sample due to the lack of spectral averaging. Nonetheless, the consistent linear decrease in reflection signal intensity across concentrations confirms the sensor substrate’s uniformity and tentative, reliable response behaviour throughout the experiment. The above results are denoted as a histogram in Figure 12c, where the 10:20 exhibited the highest cross-correlation ≈ 90% and the others ≈ 70%, respectively. The intensity value at the actual peak within a range of the SE-FTIR spectra was used to construct the calibration curve, which produced a better result than using a single maximum peak value.

**Figure 13:**
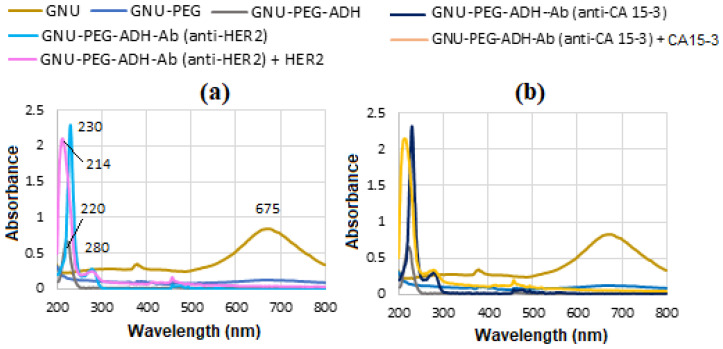
(**a**) UV–vis spectra illustrating the stepwise functionalization and bioconjugation of GNU. The initial GNU exhibits an LSPR peak at 675 nm. PEG-functionalized GNU and ADH-functionalized GNU-PEG show a peak at 220 nm, while mAb-conjugated GNU-PEG-ADH displays a peak at 230 nm. Following interaction, a characteristic peak appears at 214 nm. (**b**) Corresponding spectral results for CA 15-3.

Figure 13a displays the UV-Vis spectra captured during the stepwise functionalization and bioconjugation of GNU, which initially exhibit a LSPR peak at 675 nm. Subsequent PEG functionalization of the GNU and addition of ADH reveal a distinct absorption peak at 220 nm. Conjugation of mAb to the GNU-PEG-ADH complex yields a characteristic protein peak at 230 nm, consistent with strong amide (peptide bond) absorption typically observed between 200 and 230 nm. Following binding with the HER-II biomarker, a new absorption band emerges at 214 nm. Figure 13b is the similar corresponding results for CA 15-3. However, in these cases, CA 15-3 shows marginally higher UV-Vis absorbance than HER-II. This is likely due to CA 15-3’s comparatively larger molecular size, higher content of aromatic residues, more extensive glycosylation, and increased exposure of chromophoric regions in solution.

### 5.3. SE-FTIR of Conjugation and Interaction of BCS HER-II Samples

Recently, we reported that many overlapping FT NIR bands are associated with OH, NH, aromatic CH, carbohydrate overtones, etc., when conjugating antibodies and detecting HER-II in serum [31]. Specifically, a strong band between 6000 and 7000 cm^−1^ is assigned to OH stretch first overtones and various combinations (e.g., OH first overtones of glucose, aromatic CH first overtone). Carbohydrate biomarkers, also referred to as sugars or hydrates of carbon, are organic molecules composed of carbon, hydrogen, and oxygen (CHOs). These molecules are essential in monitoring physiological states and are widely used in the detection and management of various diseases, including diabetes, cancer, and cardiovascular disorders. Structurally, carbohydrates are categorized into several types: simple sugars such as monosaccharides (e.g., glucose, fructose) and disaccharides (e.g., sucrose); complex carbohydrates like oligosaccharides (e.g., raffinose) and polysaccharides (e.g., starch, fructans); and sugar alcohols (e.g., inositol, sorbitol, mannitol) [45,46].

**Figure 14:**
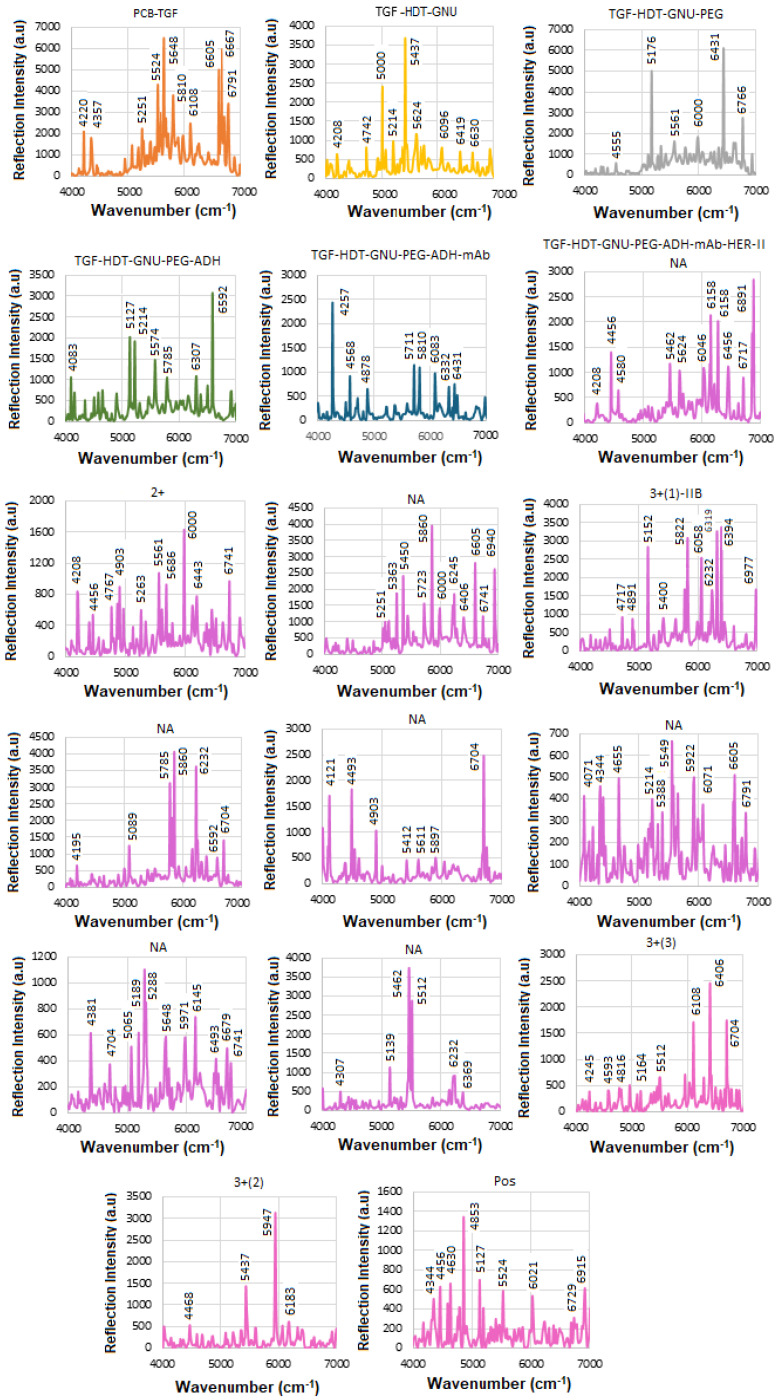
The trials of the SE-FTIR results for functionalization, conjugation, and interaction steps of the BCS HER-II biomarker.

Figure 14 shows the mAb conjugation process before and after interaction with BCS (HER-II 2+). An increase in reflection intensity signifies successful binding between the mAb and the HER-II biomarker. To improve peak visibility, the spectrum is segmented, with specific regions magnified. The following FTIR peaks have been identified: 4145 cm^−1^—Aromatic CH_2_ combination band from mAb, 4195 cm^−1^—First overtone of the OH stretch from glucose, 5102, 5139, 5164 cm^−1^—OH combination bands attributed to sucrose, glucose, and fructose, 5350, 5363 cm^−1^—RCO_2_H (carboxylic acid group) vibrations from mAb 6108 cm^−1^—First overtone of aromatic CH from mAb, 6568, 6592 cm^−1^—OH stretch overtones from glucose, and 6605, 6617, 6679 cm^−1^—Additional OH stretch overtones, possibly from glucose or similar carbohydrates. The peaks observed at 6704, 6741, and 6791 cm^−1^ are likely attributed to the first overtone of the OH stretch, possibly arising from water, alcohol groups, glucose, or other carbohydrates. These peaks may also include contributions from RNH_2_ (primary amines) or amide NH first overtones, although NH overtones are generally weaker and can overlap with OH signals. Overall, the spectral region between 6000 and 7000 cm^−1^ is rich in combination and overtone bands, particularly involving OH and CH functional groups, which are abundant in biological samples such as proteins, carbohydrates, and water [41,46,47,48,49,50,51,52].

**Figure 15:**
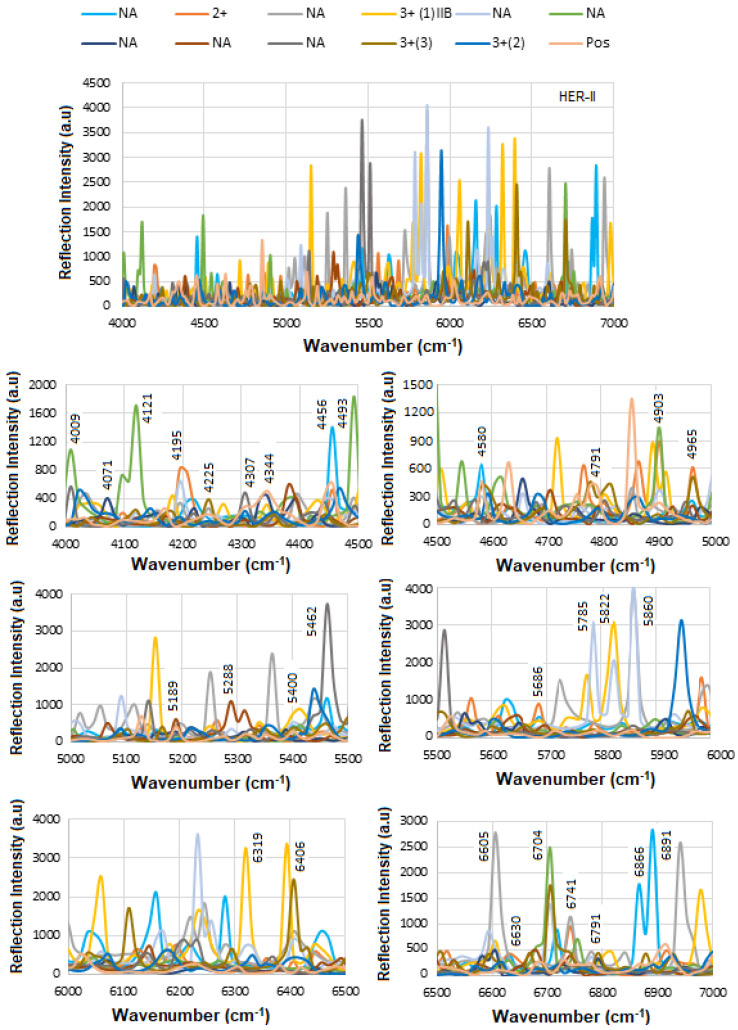
Overlapped variation in BCS-HER-II reflection intensity versus the wavenumber.

**Figure 16:**
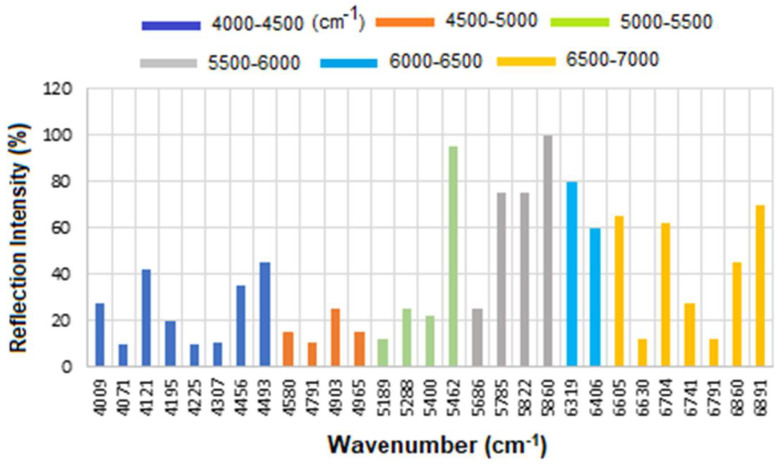
Histogram illustrating the overlapping SE-FTIR spectra of BCS HER-II within the spectral range of 4000–7000 cm^−1^.

Figure 15 shows the overlapped spectra of all concentrations for the BCS HER-II biomarkers, segmented into defined spectral regions to highlight areas with the highest degree of overlap. As previously discussed, higher concentrations lead to stronger absorption, which appears as a greater decrease in reflectance at the corresponding frequencies. Conversely, lower concentrations result in weaker absorption and therefore higher reflectance. The most significant absorption occurs in the following regions: (a) ~4000–5400 cm^−1^: Attributed to CH_2_ aromatic combinations, the first overtone of O–H stretching (from glucose), and combination or second overtone modes, (b) ~5700–6100 cm^−1^: Linked to second overtones of C–H, and the first overtone of CH_2_ stretching, mainly from lipids and proteins, and (c) ~6800–6900 cm^−1^: Corresponds to the first overtone of O–H stretching, influenced by water and hydrogen bonding dynamics. These highly overlapped spectral regions can serve as useful indicators for quantitative analysis of the BCS HER-II samples. The corresponding results are presented as a histogram in Figure 16.

**Figure 17:**
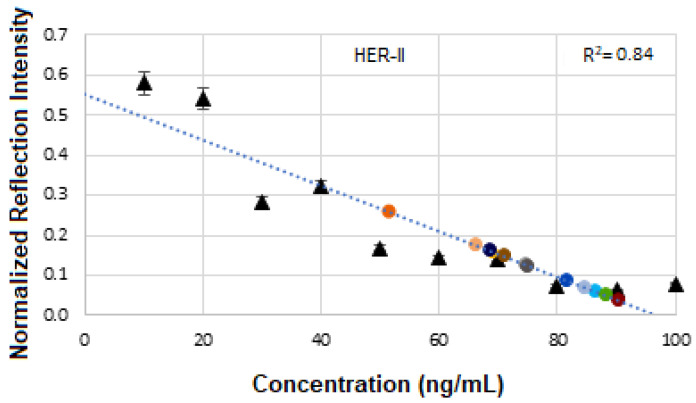
Variation in normalized intensity with BCS HER-II concentrations from the calibration curve based on experimental data used to quantify the unknown sample.

Figure 17 presents the calibration curve of normalized reflectance versus concentration, with experimental data points from unknown BCS samples plotted to determine the concentration of the corresponding HER-II stage. The linear regression yields an R^2^ value of 0.8, indicating a strong correlation between reflectance and concentration.

### 5.4. SE-FTIR of Conjugation and Interaction of BCS CA 15-3 Samples

**Figure 18:**
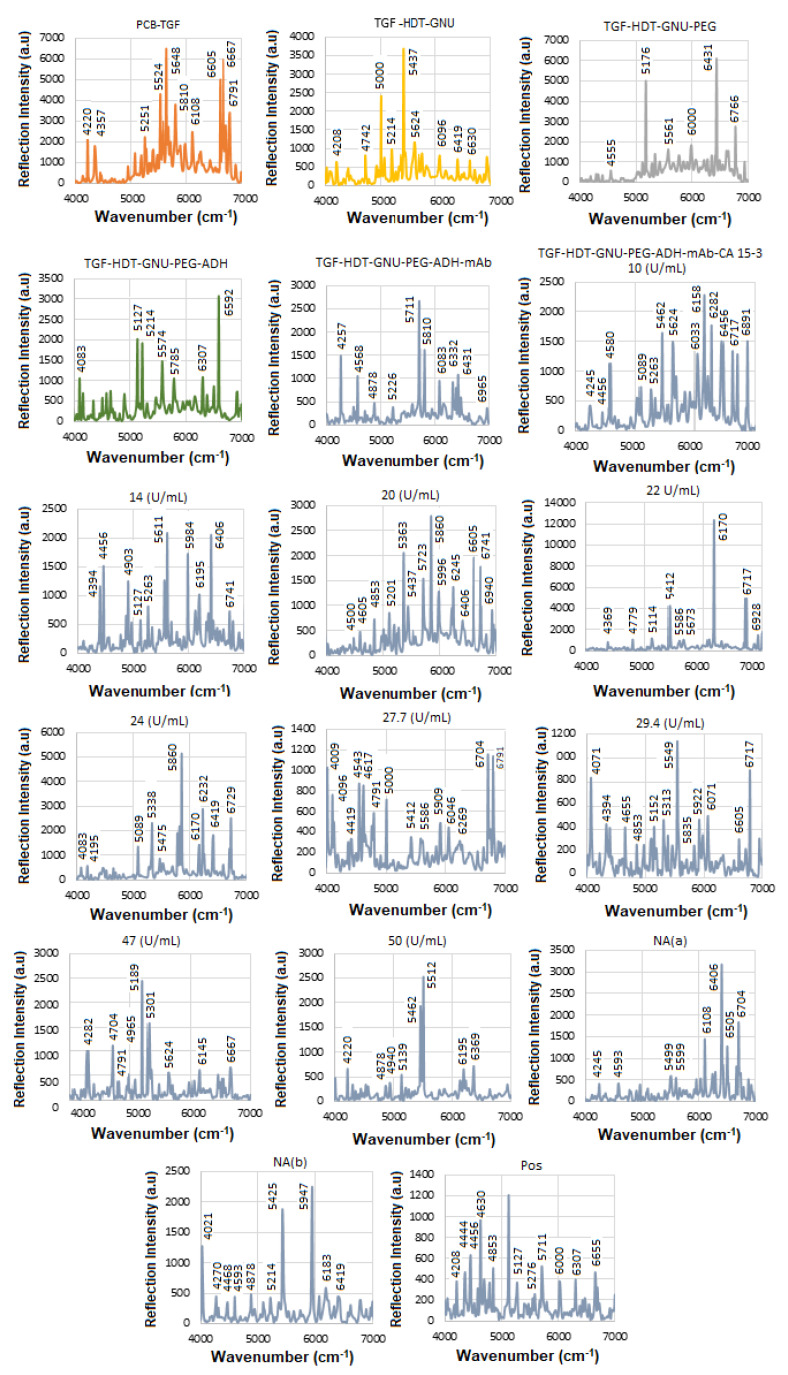
The trials of the SE-FTIR results for functionalization, conjugation, and interaction steps of the BCS CA15-3 biomarker.

**Figure 19:**
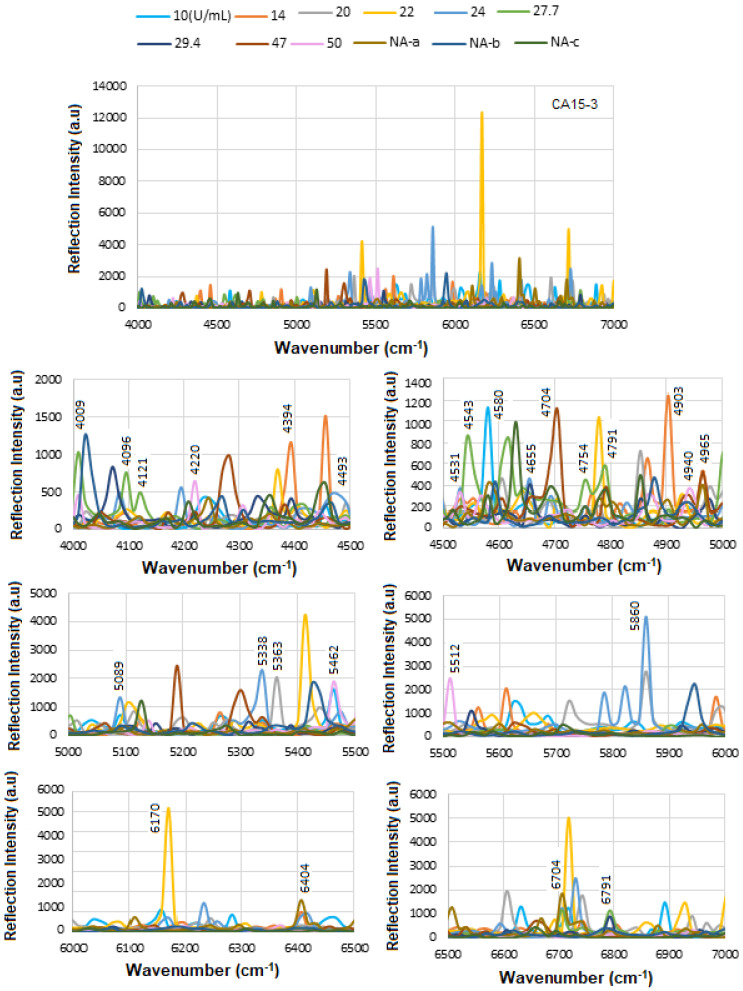
Overlapped variation in BCS-CA15-3 reflection intensity versus the wavenumber.

**Figure 20:**
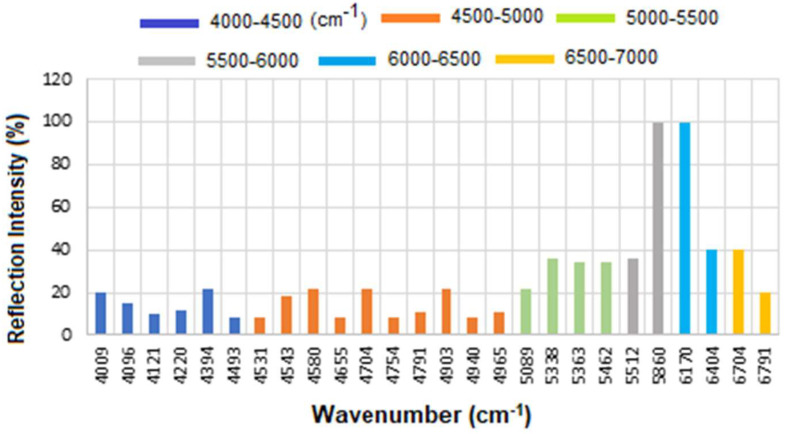
Histogram illustrating the overlapping SE-FTIR spectra of BCS CA15-3 within the spectral range of 4000–7000 cm^−1^.

Figure 18 illustrates the SE-FTIR trial results for the functionalization, conjugation, and interaction steps involving the BCS CA15-3 biomarker. The corresponding spectra are shown in a stacked format in Figure 19. To further illustrate and quantify these changes, Figure 20 presents a histogram of selected peak intensities, highlighting the spectral differences across functionalization stages. This comparative analysis helps validate each step in the bio-recognition process and confirms the sensor’s specificity and effectiveness for CA15-3 detection. The spectral analysis reveals distinct absorption features corresponding to specific molecular vibrations, which are indicative of biochemical composition. The highest absorption is observed in the range of approximately 4000–5000 cm^−1^, primarily attributed to the O–H stretching first overtone, commonly associated with glucose. This strong signal is indicative of extensive glycosylation, suggesting a high concentration of glucose or glycosylated compounds. Additionally, this region includes C–H/O–H combination bands, which are also characteristic of sugar molecules, further supporting the presence of glycosylated biomolecules.

The 5000–5500 cm^−1^ region shows relatively elevated absorption, corresponding to water, proteins, O–H combination bands typically associated with carbohydrates, and glycoproteins. The increased intensity in this region further confirms a significant contribution from carbohydrate-related structures. In contrast, the 5860–6170 cm^−1^ range, associated with the first overtone of C–H stretching in CH_2_ and CH_3_ groups, shows very low absorption. These features are commonly attributed to proteins and lipids, and the weak signal suggests a low concentration of these biomarkers in the sample or minimal lipid/protein content. Finally, the absorption increases again in the 6400–7000 cm^−1^ region, which corresponds to second overtones of O–H and N–H stretching vibrations, primarily from sugars and proteins. The relatively strong absorption in this region indicates the presence of both glycosylated sugars and possibly some protein-related structures. Overall, the spectral profile is dominated by features associated with glycosylation and carbohydrate content, while protein and lipid markers are comparatively underrepresented, suggesting a biochemical composition rich in sugar-derived functional groups.

**Figure 21:**
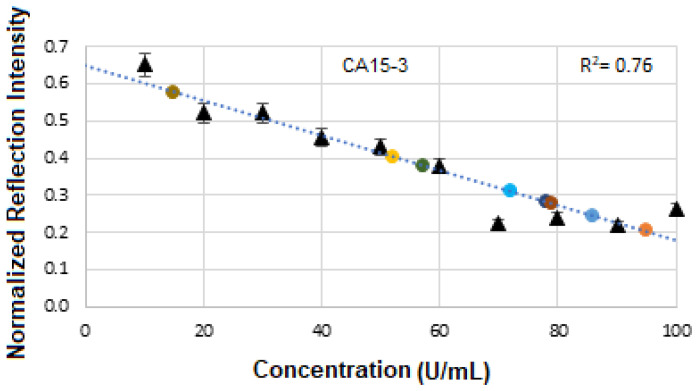
Variation in normalized intensity with BCS CA15-3 concentrations from the calibration curve based on experimental data used to quantify the unknown sample.

Figure 21 displays the calibration curve of normalized reflectance versus concentration, with experimental data points from unknown BCS samples plotted to quantify concentration levels and compare them against known reference values. The linear regression analysis produces an R^2^ value of 0.76, indicating a strong positive correlation between reflectance and concentration.

**Figure 22:**
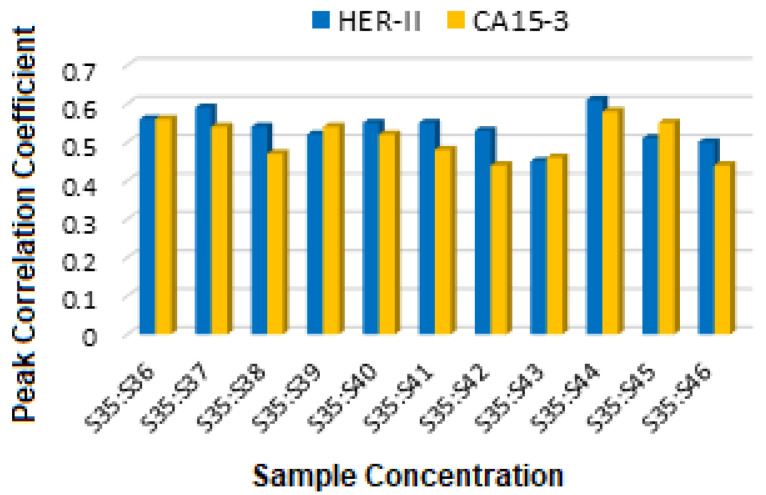
Comparison of correlation coefficient variation with the sample concentration for both HER-II and CA15-3 biomarkers.

In summary, as a comparison, the primary distinction in the spectral peak assignments between HER-II and CA15-3 biomarkers lies in the dominant vibrational modes associated with each. In the case of HER-II, the spectra are characterized by pronounced N–H and C–H overtone bands, along with protein-specific first and second overtones, reflecting the proteinaceous and glycoprotein nature of the HER-II receptor. These features are indicative of the polypeptide backbone and amine-rich side chains found in monoclonal antibodies and receptor proteins involved in HER-II expression. In contrast, CA15-3 biomarker spectra exhibit stronger absorption in regions associated with O–H stretching overtones, suggesting a higher contribution from glycosylated structures, carbohydrate moieties, or circulating mucin-type glycoproteins. These components are abundant in CA15-3, which is a soluble form of the MUC1 protein, a heavily glycosylated mucin often elevated in breast cancer patients. Overall, HER-II biomarkers show spectral features dominated by protein and amine-related overtones, whereas CA15-3 is more strongly influenced by carbohydrate-associated O–H overtones, reflecting their differing biochemical compositions and molecular structures. Figure 22 displays the comparison of the FCC between HER-II and CA15-3, where both show moderate correlation, implying partially structured biomarkers with HER-II slightly higher.

## 6. Statistical Analysis

**Figure 23:**
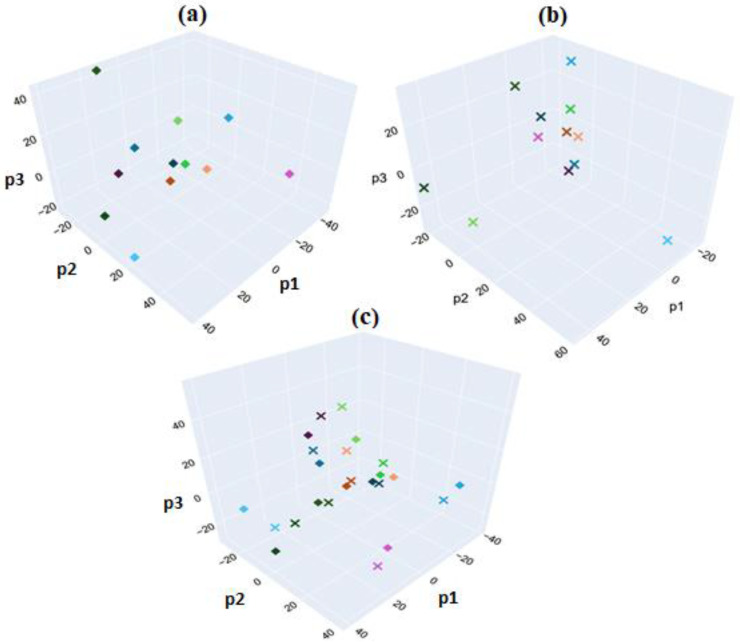
PCA discrimination of (**a**) HER-II, (**b**) CA15-3, and (**c**) combined samples. The diamonds represent HER-II samples, while crosses represent CA15-3 samples. Each colour represents a unique patient.

Partial Least Squares (PLS) regression is a dimensionality reduction technique designed to maximize the covariance between a set of independent (predictor) variables and associated dependent (target) variables (1). An advantage of PLS regression is its handling of multicollinearity, which is problematic for spectral analysis since adjacent independent variables (wavenumber reflectance intensities) can be highly correlated. PLSR transforms the original independent variables into linear combinations of fewer, uncorrelated components (latent variables). When combined with linear discriminant analysis (LDA), thresholds can be defined to enable predictive modelling based on classification. In this analysis, PLS regression was applied using the *sklearn.cross_decomposition.PLSRegression* function from *Scikit-Learn* in Python. This method was employed to assess the distinguishability between HER-II and CA15-3 biomarkers using Principal Component Analysis (PCA). In Figure 23a, PCA was applied to FTIR spectra from HER-II BCS samples, using sample numbers as target labels. Figure 23b presents a similar PCA for the CA15-3 biomarker samples. Figure 23c combines both datasets in a unified PCA, where each data point is labeled by both biomarker type and sample number, resulting in 24 unique classes corresponding to 24 distinct samples. Although this method showed limited effectiveness in distinguishing between biomarker types, it revealed a consistent underlying pattern that differentiated individual patients, as each pair of data points from the same spectrum remained closely clustered in the plot. A maximum classification accuracy of 1.0 was achieved for the biomarker samples of a given type using three PLS components.

**Figure 24:**
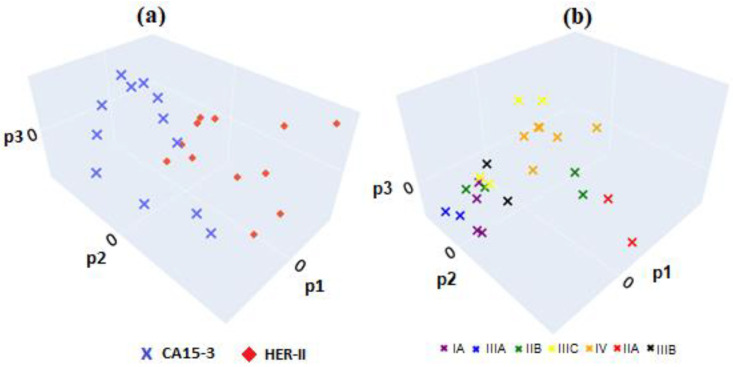
(**a**) Biomarker discrimination based on the type and (**b**) the cancer stage.

In Figure 24a, FTIR spectra from both biomarkers were used as input features, with biomarker type (HER-II or CA15-3) as the classification target. The model clearly distinguished between the two biomarker types, confirming that their FTIR spectra contain distinct and identifiable patterns. Latent variables extracted from the PLS model were used as input for LDA, and performance was evaluated using k-fold cross-validation via the *RepeatedStratifiedKFold* method in Python, with parameters set to *n_splits* = 4, *n_repeats* = 100, and *random_state* = 1. A maximum classification accuracy of 1.0 was achieved for the biomarker types using three PLS components. Principal components derived from PLS regression on the FTIR data were used to train an LDA classification model. The model was evaluated using k-fold cross-validation via Python’s *RepeatedStratifiedKFold* with parameters set to *n_splits* = 2, *n_repeats* = 100, and *random_state* = 1. In Figure 24b, the same sample set was analyzed with cancer stage (IA, IIA, IIB, IIIA, IIIB, IIIC, IV) as the classification target. Using LDA with the same cross-validation settings, a maximum classification accuracy of 0.54 was achieved for various cancer stages when using seven PLS components.

A heatmap is a data visualization tool that employs colour to represent the intensity of data points, making complex information simple to understand quickly. In research, heatmaps are most popularly used to visually summarize gene expression data across a set of samples, where hierarchical clustering can be employed to identify and investigate trends that appear in the data.

Given the size of the data collected for this research and the time-resolved nature of the datasets, a heatmap was used to quickly identify any patterns, trends, or outliers present within the data. Similarly to spectrograms, the heatmaps were generated to compare the intensities of Raman shifts across time.

**Figure 25:**
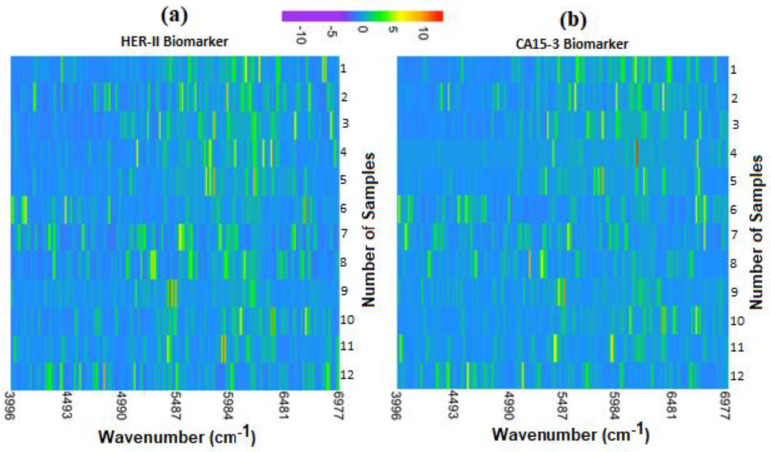
Heatmap images of (**a**) HER-II and (**b**) CA15-3 biomarkers, respectively. Each row represents a sample variation with the wavenumber.

Each row in the heatmaps represents a scan taken at given intervals (15 s for colloidal, 5 min for substrate). Each scan took 15 s to complete, so colloidal scans were collected back-to-back. Scans were z-score normalized, and intensities were visualized using a colour gradient. Figure 25 shows the heatmaps for both HER-II and CA15-3 exposing clusters of peaks between ~5000 cm^−1^ and ~6800 cm^−1^ common across most samples. This is indicated by the increased presence of green-yellow regions in this area. As investigated throughout calibration curve construction, the degree of similarity in the region may inform the quantity of CA15-3 or HER-II present in each sample.

## 7. Conclusions

Our study demonstrated that an integrated SE-FTIR spectroscopy system, combined with a PANTF-based microfluidic platform, is capable of detecting BCS biomarkers such as HER-II and CA 15-3. The EF of the system was estimated to be approximately 0.18 × 10^5^, indicating modest signal amplification relative to conventional FTIR methods. Despite the successful detection of these biomarkers, reliable quantification of unknown samples remains a challenge under the current experimental conditions. This limitation is largely attributed to batch effects in the FTIR data, arising from variability in the data acquisition process, spectrometer performance, and other instrumental factors. FCC for HER-II and CA 15-3 were consistently low, in the range of 50–55%, suggesting limited spectral similarity and weak correlation between replicate SE-FTIR datasets. Consequently, BC staging based on these biomarker signals achieved an overall classification accuracy of only 54%, which is insufficient for diagnostic applications and significantly lower than what is typically achievable using SERS-based approaches. To improve the reliability and diagnostic potential of the SE-FTIR/microfluidic platform, further optimization of the sensor design, instrumentation, and data processing pipeline is necessary. This includes minimizing batch effects, enhancing signal stability, and integrating more robust machine learning models. Moreover, expanded studies involving larger and more diverse sample sets are critical before any definitive conclusions can be drawn or comparisons made with alternative nanosensor technologies such as SERS or electrochemical detection systems.

## Figures and Tables

**Table 1:** Summary of the specifications of the BCS samples used in the experiment.

Sample No.	Specimen ID	Age	Stage Group	CA 15-3 (U/mL)	HER-II (Stage)
1	2399632	75	IA	10	-
2	2399645		IA	14	2+
3	2722228	72	IIIA	20	-
4	2372644	33	IIB	22	3+
5	HUMANSRM-0004591	36	IIIC	24.5	-
6	HUMANSRM-0118596	62	IV	27.9	-
7	HUMANSRM-0104805	41	IIB	29.4	-
8	2356746	76	IIA	47	-
9	HUMANSRM-0118596	51	IIIC	50.5	-
10	201608071	74	IIIB	N/A	3+
11	HUMANSRM-0004591	64	IV	N/A	3+
12	2572228	61	IV	N/A	POS

**Table 2:** EF values for four FTIR spectral regions.

Region (cm^−1^)	I_FTIR_ (a.u.)	I_SE-FTIR_ (a.u.)	C_FTIR_	C_SE-FTIR_	EF
~5300	0.72	1.35	10^−2^ M	10^−6^ M	0.187 × 10^5^
~5700	1.76	1.22			0.693 × 10^4^
~6000	1.43	2.08			0.145 × 10^5^
~6700	0.51	0.79			0.155 × 10^5^

**Table 3:** The tentative FTIR peak assignments to the wavenumbers.

Wavenumber (cm^−1^)	Assignments
~4021	CH_2_ aromatic combination (mAb component).
~4145	CH_2_ aromatic combination of mAb.
~4195	OH stretch first overtone of glucose.
~4606	OH bonds in the carboxyl group of PEG linker (present in the immunosensor before biomarker binding).
~4668–4719	O-H related features in the 4550–5000 cm^−1^ region (first overtones/combinations).
~5015	Combination/2nd overtone modes—OH stretch/CO stretch and also carbohydrate (sucrose/glucose/fructose) contribution.
~5102	OH combination modes of carbohydrates (s/g/f) and possibly CO stretch combination with OH/first overtone.
~5249–5333	N-H combination bans, OH first stretch overtone, RCO_2_H of mAb
~5556–6061	C-H first stretch overtone; CH_2_ first stretch overtone also in this region.
~5714–5988	C-H 2nd overtones, CH_2_ first stretch overtone
~6000–7000	OH first stretch overtones (especially of glucose), aromatic CH first overtone, and CH_2_ combination overtones.
~6108	Aromatic CH first overtone of mAb.
~6568; ~6617; ~6729	Strong OH stretch overtones of glucose (and/or RNH_2_ first overtone).

**Table 4:** The tentative CA15-3 FTIR peak assignments to the wavenumbers.

Wavenumber (cm^−1^)	Assignments	Relevance to CA15-3
~4195	OH stretch first overtone (glucose)	CA15-3 has significant glycosylation—this peak likely appears.
~5015	CH/OH combinations (sugars)	Again, due to glycosylation, this region should show peaks.
~5100–5300	OH first overtone, O-H combination bands (carbohydrates), RCO_2_H, CH combinations	Broad absorption bands likely present.
~5550–6000	CH_2_/CH_3_ first stretch overtones (proteins/lipids)	Expected due to peptide backbone and lipid components.
~6000–7000	OH and CH overtones	Common across many glycoproteins—present in CA15-3 too.
~6560–6730	OH and NH overtones (from sugar and protein)	Likely visible depending on structural conformation and sample prep.

## Data Availability

The original contributions presented in this study are included in the article. Further inquiries can be directed to the corresponding author.
